# Extending Cofactor‐Tethered Nanomachines to Complex Multienzyme Redox Cascades in Continuous Flow

**DOI:** 10.1002/cssc.70914

**Published:** 2026-07-24

**Authors:** Zinnia Dsouza, Jan‐Simon Jeshua Friedrichs, Okke Melse, Gerhard Schenk, Volker Sieber

**Affiliations:** ^1^ Chair of Chemistry of Biogenic Resources Campus Straubing for Biotechnology and Sustainability Technical University of Munich Straubing Germany; ^2^ School of Chemistry and Molecular Biosciences The University of Queensland St. Lucia Australia

**Keywords:** continuous flow biocatalysis, modified nicotinamide cofactors, nanomachines, redox cascades

## Abstract

Multienzyme cascades are emerging as alternatives to fermentation for converting biogenic feedstocks into value‐added chemicals and fuels. A major bottleneck in redox transformations is the loss of costly and unstable cofactors in continuous‐flow systems, typically necessitating co‐immobilization and complex regeneration schemes. Self‐sufficient biocatalytic nanomachines, created by fusing enzymes with tethered cofactors, offer a streamlined solution by enabling intramolecular cofactor retention and recycling. Here, we demonstrate that such nanomachines can be integrated into a complete redox‐dependent cascade, exemplified by a 10‐step glucose‐to‐isobutanol pathway. Oxidoreductases were fused into redox pairs using peptide linkers containing a single cysteine for covalent attachment of polyethylene glycol (PEG)‐modified nicotinamide cofactors, with rational selection of monomeric enzymes ensuring structural compatibility and soluble expression. The resulting nanomachines remained catalytically competent and produced isobutanol in both batch and continuous‐flow operation without the addition of free NAD^+^. A total turnover number of ∼1.4 × 10^4^ was achieved for tethered PEG–NAD^+^, among the highest reported for immobilized nicotinamide cofactors. Use of the ultrastable analogue PEG–cNAD^+^ further demonstrates the modularity of the platform. Together, these results establish a proof‐of‐concept for cofactor‐autonomous multienzyme redox cascades in continuous flow and define design principles for future cell‐free synthesis systems.

## Graphical abstract

Engineered enzyme fusions bearing covalently tethered PEG–nicotinamide cofactors enable intramolecular cofactor retention and recycling within self‐sufficient biocatalytic nanomachines. These nanomachines support cofactor‐autonomous operation of complex redox multienzyme cascades under continuous‐flow conditions, as demonstrated in a 10‐step glucose‐to‐isobutanol transformation.

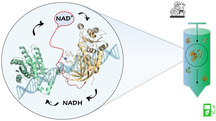

## 1 Introduction

Biocatalysis is rapidly expanding beyond pharmaceuticals into large‐scale production of platform chemicals and bulk intermediates. A central driver is the conversion of biogenic feedstocks into value‐added chemicals and fuels using multienzyme cascades, which emulate natural stepwise catalysis while offering (i) high atom economy, (ii) reduced waste, and (iii) operational flexibility in batch and continuous‐flow systems [1, 2, 3]. Compared with microbial fermentation, cell‐free enzyme systems bypass cellular regulation, improve yields, and allow finer control of conditions and kinetics [4]. However, industrial application is limited by practical challenges. Strategies to improve performance include reducing side products, enhancing substrate channeling, stabilizing intermediates, shifting equilibria in favour of products, eliminating inhibition, and optimizing enzyme stoichiometry and spatial organization [5, 6].

Designing effective cascades requires the balancing of catalytic efficiency, selectivity, stability, and process integration. Industrial deployment is often constrained by reliance on multiple purified enzymes and expensive diffusible cofactors [7], but upstream expression and purification costs are often omitted from sustainability assessments [8, 9]. Redox‐intensive cascades face additional challenges because NAD(P)H cofactors are consumed stoichiometrically and typically regenerated using sacrificial substrates, yet also suffer chemical degradation during turnover [10, 11]. True cofactor recycling refers to redox‐state regeneration, whereas irreversible losses arise from chemical degradation of NAD(P)^+^/NAD(P)H. In vivo, salvage pathways restore cofactors [12], and while also applicable in vitro [13], in vitro systems typically require costly replenishment. Three core limitations thus emerge: (i) cofactor instability, (ii) poor retention, and (iii) incompatibility with continuous‐flow operation. Freely diffusing cofactors are especially problematic under flow, where washout, dilution, and uncontrolled diffusion disrupt synchronized catalysis and promote side reactions [14, 15].

To address these limitations, a variety of strategies have been developed, including integrated cofactor recycling modules [16], enzyme immobilization [17], co‐immobilization of enzymes and cofactors [18], and spatial organization through programmable materials, co‐encapsulation [19], and compartmentalization [20, 21]. However, most implementations continue to rely on auxiliary recycling enzymes and freely diffusing cofactor pools, leaving challenges of cofactor instability, loss, and process integration largely unresolved. Covalent attachment of chemically modified cofactors has been explored as a means to improve cofactor retention and reduce leaching [22, 23], but such approaches have rarely been incorporated into autonomous, multistep catalytic systems. Although the broader challenges of cofactor regeneration have been extensively reviewed [24], strategies that simultaneously address cofactor instability, retention, and compatibility with continuous‐flow operation remain limited. These constraints are exacerbated in continuous‐flow reactors, where unidirectional transport disrupts cofactor retention and synchronization, leading to washout, dilution, and cumulative degradation [25]. Various strategies for cofactor immobilization have been developed to facilitate continuous‐flow biocatalysis and have been extensively reviewed [17]. However, their implementation within complex multienzyme redox cascades operating under continuous‐flow conditions remains rare. A compelling strategy is the development of self‐sufficient biocatalysts that retain and recycle cofactors internally. Hartley *et al*. introduced proof‐of‐concept “biocatalytic nanomachines” by tethering semisynthetic cofactors to engineered enzymes, enabling cofactor retention and intramolecular recycling under continuous‐flow conditions [26]. By anchoring cofactors proximal to catalytic sites, these systems minimize leaching, maintain redox balance, and enhance catalytic performance.

Building on this framework, we extended the nanomachine concept to a fully integrated multienzyme redox cascade, i.e., the conversion of D‐glucose to isobutanol, which requires four redox transformations [27]. Following the “biocatalytic nanomachine” design, we engineered complexes comprising catalytic, recycling, and immobilization modules to enable site‐specific attachment and cofactor retention [26]. Module selection and fusion architecture were informed by structural compatibility, terminus accessibility, and enzyme oligomeric state, enabling site‐specific cofactor attachment and complex assembly without compromising native folding or catalytic activity. The nanomachines were validated in batch and continuous flow, and total turnover numbers (TTN) for tethered cofactors were quantified. Site‐specific modification also enabled incorporation of a thermostable synthetic cofactor, carba‐NAD^+^ (cNAD^+^) [28, 29]. Collectively, these results define a versatile design principle for compact, self‐sufficient redox biocatalysts and highlight the potential of cofactor‐autonomous multienzyme systems for synthetic biology, flow biocatalysis, and sustainable chemical manufacturing.

## 2 Results and Discussion

### 2.1 Extending Nanomachine Design to a Multienzyme Redox Cascade

**FIGURE 1:**
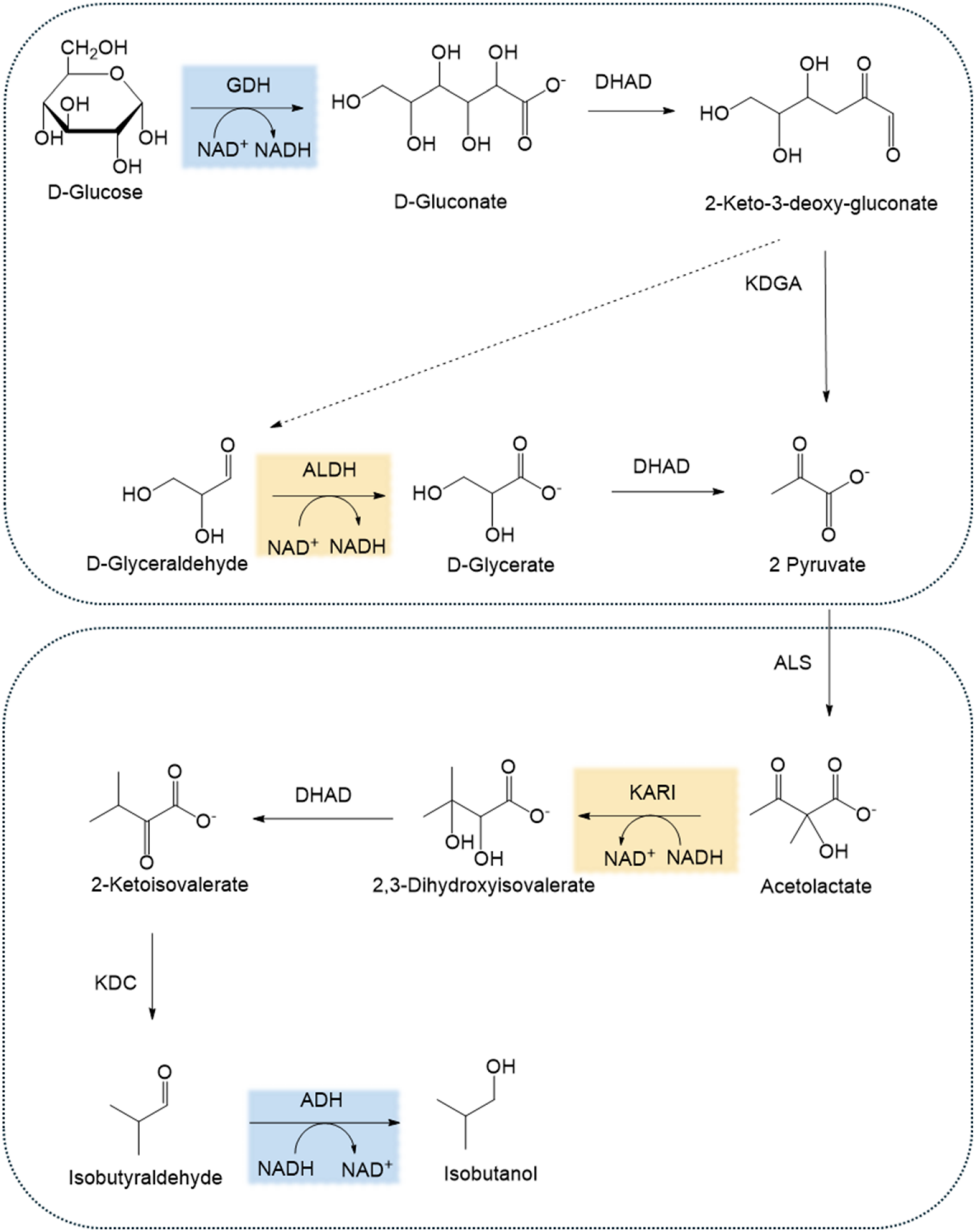
Schematic representation of the multienzymatic cascade for the conversion of D‐glucose to isobutanol. The pathway comprises 10 consecutive reactions catalyzed by eight enzymes and is organized into two functional modules: (i) a glycolytic module that converts glucose to pyruvate and (ii) an isobutanol module that transforms pyruvate into isobutanol.

To test whether the biocatalytic nanomachine concept could be extended to complex transformations, we implemented it in the 10‐step enzymatic conversion of D‐glucose to isobutanol, marking the first integration of nanomachines into a multienzymatic redox network (Figure 1). This cascade can direct flux toward either ethanol or isobutanol [27]; here, we focused exclusively on the isobutanol branch.

The pathway was organized into two interconnected functional modules. The first module, the “pyruvate module,” funnels glucose into pyruvate via four enzymes, i.e., glucose dehydrogenase (GDH), a dehydratase (DHAD), an aldolase (KDGA), and an aldehyde dehydrogenase (ALDH), and a single NAD^+^ cofactor. One glucose molecule is converted into two pyruvate molecules while reducing NAD^+^ to NADH, efficiently channeling carbon into the next stage. The second module, the “isobutanol module,” converts pyruvate into isobutanol through four additional enzymes. Acetolactate synthase (ALS) condenses pyruvate into acetolactate, which is processed by ketol‐acid reductoisomerase (KARI) and another DHAD to ketoisovalerate; a ketoacid decarboxylase (KDC) then produces isobutyraldehyde, and an alcohol dehydrogenase (ADH) yields the final isobutanol product. Together, the modules contain four redox reactions, requiring efficient NAD^+^/NADH cycling.

A central design feature is that both modules rely exclusively on NAD^+^/NADH as the sole redox cofactor, simplifying cofactor management and reducing operational complexity, and that no other cosubstrate (i.e., ATP/ADP) is required. Moreover, the modularity of the four oxidoreductases (GDH, ALDH, KARI, and ADH) allows their pairing into discrete oxidative–reductive steps, ideally suited for nanomachine assembly. In contrast to prior nanomachine designs that relied on auxiliary recycling enzymes, we leveraged the intrinsic redox neutrality of the cascade to construct two nanomachines, each combining one oxidative and one reductive transformation (Figure S1).

### 2.2 Rational Design and Construction of Nanomachines

#### 2.2.1 Choice of Enzyme Candidates and Redox Pairs

We selected enzyme variants for the glucose‐to‐isobutanol cascade based on a combination of catalytic performance, substrate selectivity, and structural suitability for fusion (Table S1). The first step, glucose oxidation, is catalyzed by GDH [27]. Although the previously utilized *Sulfolobus solfataricus* GDH (*Ss*GDH) is highly thermostable, its low turnover under mild conditions limits efficiency. To balance stability and activity, we chose a *Bacillus subtilis* GDH (*Bs*GDH) double mutant, engineered to retain thermostability while providing substantially higher catalytic rates [30, 31]. This ensures sufficient activity even after fusion. For aldehyde oxidation, the *Thermoplasma acidophilum* ALDH (*Ta*ALDH M42) previously employed in this cascade exhibited limited substrate selectivity, causing side reactions. Comparison with an engineered *Herbaspirillum seropedicae* ALDH (*Hs*ALDH), optimized for glyceraldehyde, revealed superior catalytic efficiency and pathway fidelity (Table S1) [32]. Consequently, *Hs*ALDH was selected. The reduction steps were catalyzed by KARI from *Meiothermus ruber* (*Mr*KARI) and the ADH from *Geobacillus stearothermophilus* (*Bst*ADH), which have previously been used in the isobutanol cascade [27]. To ensure compatibility for fusion, we analyzed enzyme topology in silico, including overall structure, orientation of catalytic sites, and N‐ and C‐terminal accessibility (Table S2). Combining these considerations of activity, selectivity, and structural feasibility, we established two NAD^+^/NADH‐dependent redox pairs for nanomachine construction: (1) GDH fused to ADH, and (2) KARI paired with ALDH. These four oxidoreductases were modularly assembled into two nanomachines, each coupling an oxidative and a reductive transformation, enabling internal cofactor recycling and streamlined cascade operation.

#### 2.2.2 Assembly and Functional Evaluation of Fusion Constructs

Nanomachine fusion proteins were designed using a modular strategy adapted from Hartley et al. [26]. Each construct comprised two NAD^+^‐dependent oxidoreductases joined by a flexible 19‐amino‐acid glycine‐serine (GSS) linker. A single cysteine residue was positioned centrally within the linker to enable site‐specific attachment of a maleimide‐functionalized, polyethylene glycol (PEG)‐modified NAD^+^ cofactor via thiol–maleimide conjugation. All constructs carried an N‐terminal hexahistidine tag to facilitate metal‐affinity purification and to enable site‐specific immobilization in continuous‐flow applications, differing from the earlier nanomachine design [26], which relied on an additional conjugation module (esterase domain) for covalent attachment to TFK‐activated agarose beads. Fusion genes were assembled by PCR amplification of the individual enzyme coding sequences with overlapping extensions encoding the linker, enabling seamless in‐frame cloning into the expression vector. To systematically probe the influence of domain order on expression, folding, and catalytic performance, all four possible configurations were generated and evaluated (N‐terminus to C‐terminus): GDH–ADH, ADH–GDH, ALDH–KARI, and KARI–ALDH.

#### 2.2.3 Expression and Catalytic Evaluation of the Fusion Constructs Using Free Cofactor

Although all fusion constructs were successfully assembled, their expression, solubility, and catalytic activity varied substantially. Both fusion constructs, irrespective of their configurations, exhibited low expression levels, poor solubility, and a pronounced loss of activity relative to their free enzyme counterparts (Figure S3a,b), consistent with structural instability and misfolding. Attempts to restore activity by modifying linker length (ranging from nine to 33 amino acids in length) were unsuccessful, as none of the tested variants yielded stable, active proteins (data not shown). Further analysis identified the oligomeric state of the constituent enzymes as a primary determinant of fusion feasibility. Size‐exclusion chromatography of the poorly performing constructs revealed multiple elution peaks, indicative of heterogeneous higher‐order assemblies and improper folding (Figure S3c). Consistent with previous reports [33], characterization of the free enzymes showed that most exist as oligomers, typically tetramers, while *Mr*KARI assembles as a dodecamer. Such high‐order oligomerization is intrinsically unfavorable for fusion protein design, as it exacerbates steric constraints at subunit interfaces and increases susceptibility to misfolding [34].

To overcome this limitation, we shifted our strategy toward monomeric oxidoreductases. Screening identified two suitable candidates: an ADH from *Thermococcus kodakarensis* (*Tk*ADH) [35] and a KARI from *Ignisphaera aggregans* (*Ia*KARI), the latter available in monomeric form as a result of prior protein engineering [36].

New fusion constructs were generated by substituting the oligomeric enzymes with their monomeric counterparts (*Tk*ADH replacing *Bst*ADH and *Ia*KARI replacing *Mr*KARI). These redesigned fusions showed markedly improved soluble expression and higher relative activities compared to the original constructs (Figure S4), consistent with enhanced folding and stability. Among the redesigned constructs, the ADH–GDH and KARI–ALDH configurations produced the highest yields of soluble fusion protein (Figure S5a,b), while retaining at least ∼50% of the specific activity of the corresponding free enzymes (Figure S4). In contrast, the GDH–ADH configuration expressed poorly, formed heterogeneous oligomeric species (monomers and hexamers) (Figure S6), and displayed substantially reduced activity; structural modeling implicated the extended C‐terminal loop of *Bs*GDH as a likely contributor to misfolding in this orientation (Table S2). Similarly, the ALDH–KARI configuration was soluble but lacked detectable KARI activity, indicating domain‐level structural incompatibility. Importantly, in the efficient configurations (i.e., ADH–GDH and KARI–ALDH), the catalytic turnover numbers (*k*_cat_) of the individual enzyme domains were preserved or, in some cases, enhanced relative to the free enzymes (Table 1), demonstrating that rational fusion did not compromise intrinsic catalytic performance.

**TABLE 1:** Kinetic parameters of oxidoreductases in free, fused and cofactor‐tethered configurations.

Enzyme	***V***_**max**_ **, ** **U/mg**	***k***_**cat**_ **, ** **s** ^**−1**^	***K***_**M**_ **substrate, mM**	***K***_**M**_ **cofactor, mM**
Glucose dehydrogenase (GDH)				
*Bs*GDH	173 ± 2	87	3.0 ± 0.2	0.90 ± 0.01
*Tk*ADH‐*Bs*GDH	135 ± 6	142	5.7 ± 0.9	0.90 ± 0.05
*Tk*ADH‐PegNAD^+^‐*Bs*GDH	67 ± 2	71	6.0 ± 0.5	N/A
Alcohol dehydrogenase (ADH)				
*Tk*ADH	10.4 ± 0.3	5.9	14 ± 1	0.25 ± 0.05
*Tk*ADH‐*Bs*GDH	8.2 ± 0.1	8.7	13 ± 2	0.25 ± 0.02
Glyceraldehyde dehydrogenase (ALDH)				
*Hs*ALDH	78 ± 7	69	3.1 ± 0.6	0.40 ± 0.01
*Ia*KARI‐*Hs*ALDH	59 ± 2	106	6.2 ± 0.8	0.80 ± 0.06
*Ia*KARI‐PegNAD^+^‐*Hs*ALDH	17 ± 1	31	10.5 ± 2.5	N/A
Ketol acid reductoisomerase (KARI)				
*Ia*KARI	1.40 ± 0.04	1.23	3.3 ± 0.3	<0.1 mM
*Ia*KARI‐*Hs*ALDH	1.00 ± 0.03	1.80	0.37 ± 0.06	<0.1 mM

*Notes*: Michaelis–Menten constants (*K*_M_), turnover numbers (*k*_cat_ *)*, and *V*_max_ were determined for free enzyme forms, fusion constructs, and nanomachines carrying covalently tethered PEG24–NAD^+^. For nanomachines, kinetic parameters were determined for the oxidative domains (GDH and ALDH) by monitoring formation of the reduced tethered cofactor at 340 nm. Because PEG–NAD^+^ was covalently tethered and not varied during the assay, kinetic parameters were determined only for the respective substrate. Data are reported as means ± SD from at least three independent measurements.

Abbreviation: N/A, not applicable.

A clear trend emerged in which positioning the monomeric enzyme at the N‐terminus favored proper cotranslational folding and improved solubility. Accordingly, ADH–GDH and KARI–ALDH were predominantly monomeric in solution, as confirmed by size‐exclusion chromatography (Figure S5c,d), and were therefore selected for further functional characterization. Each construct eluted as a single peak with an apparent molecular mass close to the calculated monomeric mass, consistent with monomeric behavior under the conditions tested. This outcome was unexpected, as the C‐terminal domains could, in principle, still mediate oligomerization; however, fusion likely perturbs or sterically occludes native subunit interfaces, particularly at the C‐termini, which are either engaged in the peptide linkage or shielded by the fused partner.

#### 2.2.4 Evaluation of Thermal Stability

**FIGURE 2:**
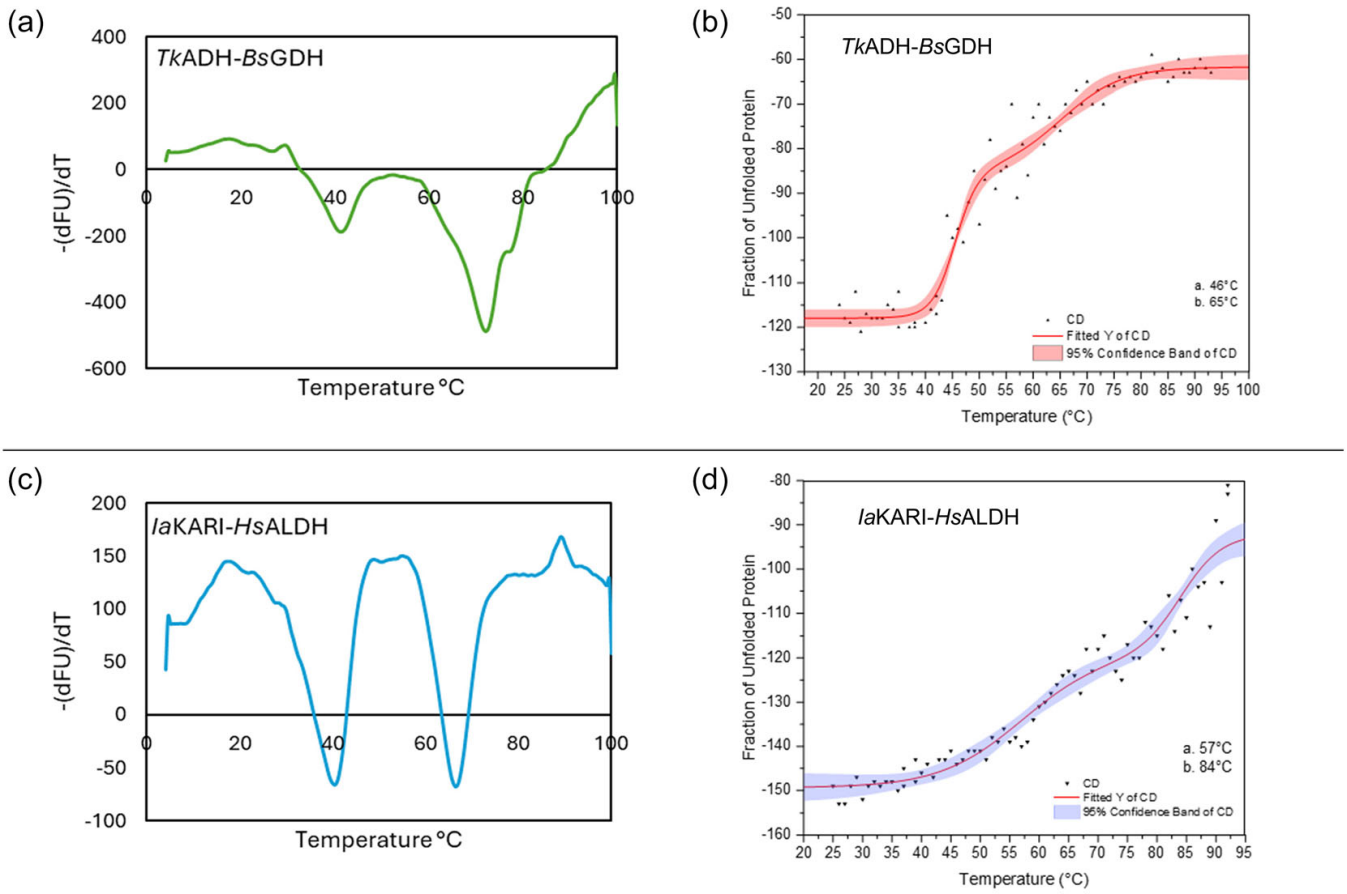
Thermal unfolding of fusion constructs. (a,b) Thermal stability of *Tk*ADH–*Bs*GDH monitored by thermofluor (a) and (b) CD spectroscopy. (c,d) Thermal stability of *Ia*KARI–*Hs*ALDH monitored by thermofluor (c) and (d) CD spectroscopy. Both fusion constructs exhibit biphasic melting transitions, consistent with sequential unfolding of the two domains.

We next evaluated the thermal stability of the optimized fusion constructs, as all individual enzyme components exhibit high intrinsic thermostability in their free form (*Bs*GDH: 77.5 °C, *Tk*ADH: >100 °C, *Hs*ALDH: 63 °C, *Ia*KARI: 94 °C) (Figure S7). Thermal unfolding was assessed using thermofluor assays and circular dichroism (CD) spectroscopy. For both fusion proteins (ADH–GDH and KARI–ALDH), unfolding profiles were biphasic, consistent with sequential denaturation of the two fused domains (Figure 2). In both cases, the apparent melting temperatures were markedly reduced relative to those of the corresponding free enzymes. For the *Tk*ADH–*Bs*GDH fusion, CD analysis revealed unfolding transitions at ≈46 and 65 °C, while the *Ia*KARI–*Hs*ALDH fusion displayed transitions at ≈57 and 84 °C. Thermofluor analysis gave values of 42 and 72 °C for *Tk*ADH–*Bs*GDH and 41 and 67 °C for *Ia*KARI–*Hs*ALDH. Although the absolute transition temperatures differed between thermofluor and CD measurements, reflecting the distinct sensitivities of the two techniques (thermofluor indicating opening of the structure and revealing hydrophobic regions versus CD showing changes in secondary structure), both methods consistently indicated substantial destabilization upon fusion. These data establish an upper temperature boundary for subsequent experiments and informed the selection of operating conditions well below the lowest unfolding transition.

The reduced thermal stability of the fusion constructs is most likely attributable to interdomain effects introduced by the monomers being forced together via the covalent linkage. Importantly, reduced thermal stability did not preclude robust catalytic activity under the assay and operational conditions used in this study. In the monomeric fusion context, oligomerization via native C‐terminal interfaces is suppressed, eliminating stabilizing interactions normally provided by multimerization. Upon heating, unfolding of the fusion proteins appears to proceed cooperatively: strain imposed by the linker and constrained domain–domain interactions might promote early exposure of hydrophobic regions, while secondary structural elements remain transiently intact [37, 38]. Consequently, both domains unfold at lower temperatures than in their free, oligomeric states, indicating mutual destabilization within the fusion architecture. Thus, the loss of native oligomeric interfaces and the introduction of interdomain constraints act synergistically to reduce the overall thermal stability of the nanomachine constructs. Nevertheless, the residual stability is fully compatible with their intended operational window, demonstrating that structural integration can be achieved without sacrificing functional robustness.

### 2.3 Structural Modeling to Define Optimal PEG Linker Length

**FIGURE 3:**
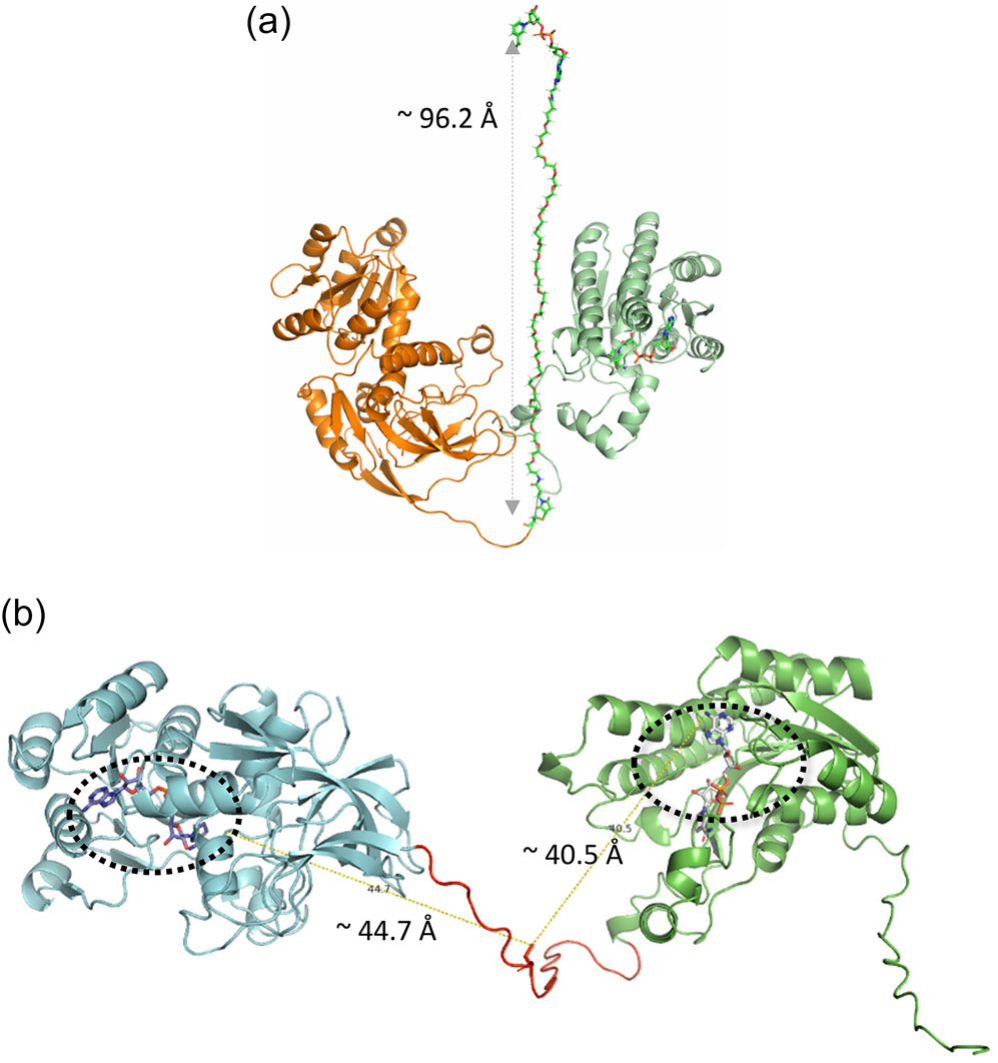
Structural modeling of PEG24–NAD^+^ tethering in fusion constructs. (a) Alphafold‐predicted structure of a representative fusion enzyme: ADH‐GDH with PEG24–NAD^+^ modelled as a flexible tail extending from the engineered cysteine in the Gly–Ser linker. The PEG24 linker has a calculated contour length of ∼96.2 Å, sufficient to reach NAD^+^‐binding sites. (b) Alphafold model of the same fusion construct with NAD^+^‐binding pockets highlighted and distances from the central cysteine to each site annotated.

To determine the optimal PEG chain length for intramolecular cofactor transfer and ensure efficient access of the tethered cofactor to both enzyme active sites, we conducted in silico modeling using AlphaFold‐predicted structures of the fusion constructs in their monomeric state. The engineered cysteine residue within the Gly–Ser linker, which serves as the conjugation site for maleimide‐PEG‐NAD^+^, was estimated to lie ∼10.6 Å from the PEG attachment point (Figure S1d). Based on a 10‐mer model generated in Maestro (Schrödinger, LLC), each PEG repeat unit was approximated to extend the linker by ∼3.6 Å. This yielded contour lengths of ∼53.4 Å for PEG12, ∼96.2 Å for PEG24, and ∼181.9 Å for PEG48. Structural analysis indicated that the distance from the central cysteine to the NAD(H)‐binding pockets of the partner enzymes ranged from ∼40–65 Å, depending on enzyme orientation (Figure 3). In addition, molecular dynamics simulations of the fusion construct in the apo state (i.e., in the absence of the PEG–NAD^+^ conjugate) revealed largely independent motions of the two enzyme domains connected by the flexible linker, while preserving the structural integrity of each catalytic domain (Figure S2). Consistent with these estimates, visual inspection of the structural models indicated that PEG24 provides sufficient reach to bridge the inter‐domain separation without introducing excessive linker length or undue conformational freedom. Additionally, the Gly–Ser linker itself was predicted to confer sufficient domain independence, enabling relative movement of the fused enzymes and supporting efficient cofactor shuttling.

### 2.4 Functional and Chemical Validation of Intramolecular Cofactor‐Tethered Nanomachines

Following successful construction and catalytic characterization of the fusion constructs (Figure S3), we engineered modified cofactors for covalent tethering, enabling fully self‐sufficient enzymatic activity. Both natural NAD^+^ and the synthetic analogue cNAD^+^ [28, 39] were derivatized at the C6‐amine of the adenine ring and coupled to a maleimide‐activated PEG24 linker (informed by in silico modeling) to produce mal‐PEG24‐N6‐NAD^+^ and mal‐PEG24‐N6‐cNAD^+^ (Figure S8). Conjugation was achieved via the central cysteine in the flexible Gly–Ser linker connecting the enzyme domains. The resulting tethered nanomachines (e.g., *Tk*ADH‐mal‐PEG24‐N6‐NAD^+^‐*Bs*GDH) demonstrated robust catalytic activity without the need for externally added cofactor, highlighting successful intramolecular cofactor transfer. Kinetic analyses (*K*_m_ and *k*_cat_ values) of the oxidation partners GDH and ALDH confirmed efficient cofactor shuttling within the nanomachines (Table 1). Covalent attachment was further validated by quantifying accessible thiols before and after conjugation using Ellman's reagent. The absence of a colorimetric signal confirmed complete consumption of free cysteines, consistent with full cofactor tethering (Figure S9).

### 2.5 Performance of Tethered Nanomachines in the Redox Cascade Under Batch and Continuous Flow

To assess the activity of tethered nanomachines within a full multienzymatic network, the fusion constructs were integrated into the complete glucose‐to‐isobutanol cascade and evaluated under both batch and continuous‐flow conditions. For flow experiments, the N‐terminal His tags, originally introduced for immobilized metal affinity chromatography (IMAC) purification, were repurposed as immobilization handles, avoiding additional chemical/genetic modifications that could compromise enzyme activity. Nanomachines were immobilized on 1 mL His GraviTrap columns (∼40 mg mL^−1^ binding capacity) at subsaturating loading. Following cofactor tethering, the nanomachines were combined with the remaining enzymes of the 10‐step cascade (Table S4 and Figure S10) at stoichiometries adapted from previous studies [32, 40] and applied as an enzyme mixture to the column (15.68 mg mL^−1^ of total enzyme). Successful immobilization was confirmed by monitoring the UV absorbance of the column flow‐through at 280 nm, which showed no detectable protein. In parallel, batch reactions were performed in closed microtubes under identical conditions with 100 mM glucose as substrate, enabling direct comparison of nanomachine performance between freely diffusing and immobilized formats. Isobutanol formation (vide infra) was monitored by high‐performance liquid chromatography (HPLC) time‐course analysis in both systems to assess cascade functionality under batch and continuous‐flow operation. Under these conditions, tuning the ratios of fusion enzymes (relative activities) was inherently constrained by the fixed 1:1 stoichiometry imposed by genetic linkage. Given the substantial differences in intrinsic catalytic activities between the paired domains (e.g., ∼16‐fold for *Tk*ADH–*Bs*GDH and ∼60‐fold for *Ia*KARI–*Hs*ALDH), the slower domain dictated the local turnover rate of the associated reaction pair, thereby setting the pace of intermediate flux and cofactor recycling within corresponding segments of the cascade. In contrast to systems employing separate enzymes, where individual component concentrations can be independently adjusted, the fusion format precludes such optimization, as both activities are obligatorily covaried. Consequently, the slower domain sets the pace of intermediate flux and cofactor recycling within each segment of the cascade, highlighting a fundamental limitation of fixed‐stoichiometry fusion designs.

#### 2.5.1 Substrate Conversion and Isobutanol Titers

**FIGURE 4:**
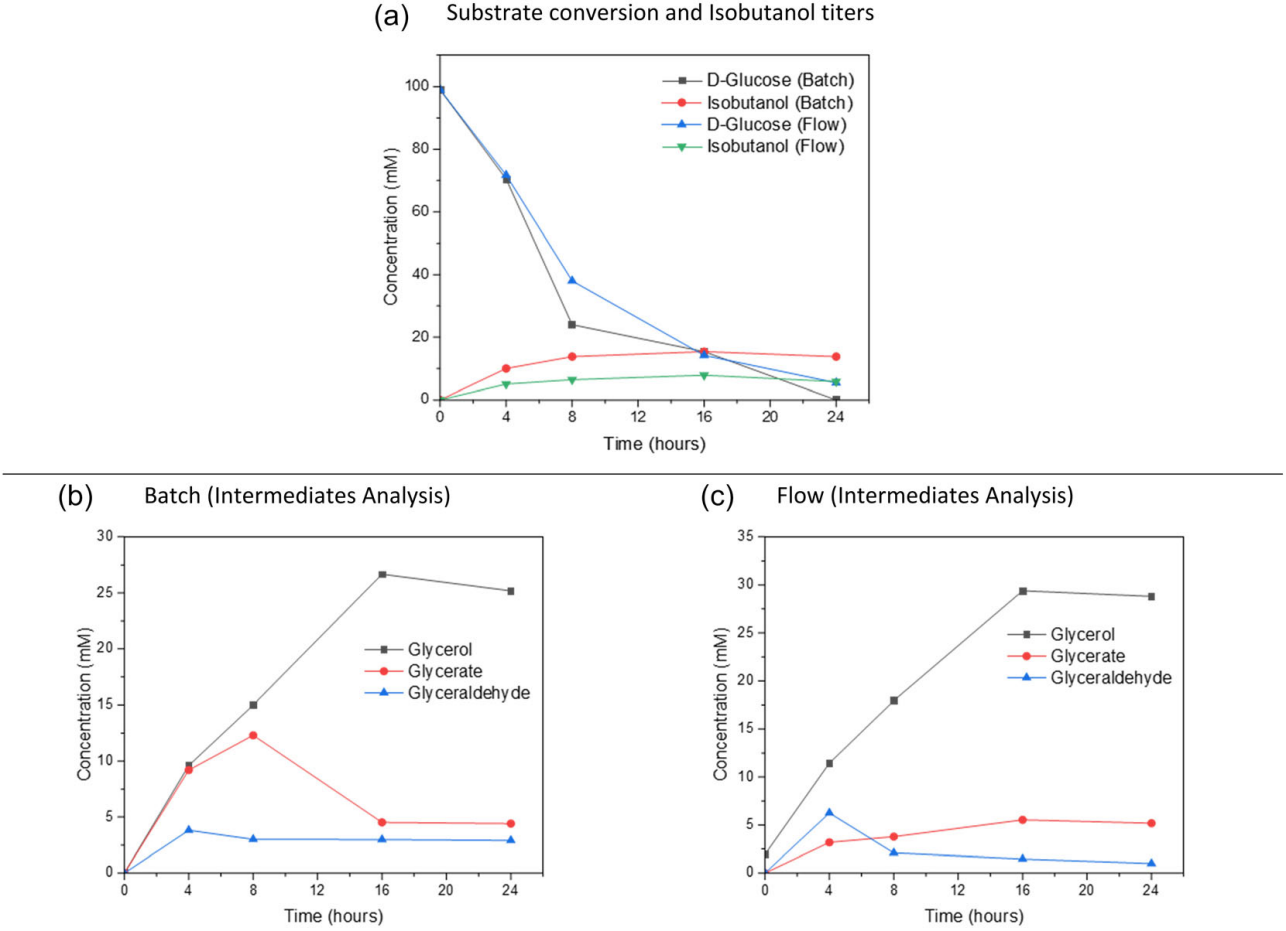
Substrate conversion, product formation, and intermediate accumulation in cascades run in batch and flow modes. (a) Glucose consumption and isobutanol production over 24 h, showing complete conversion in batch mode (13.8 mM isobutanol) but slower, incomplete conversion in flow mode (peak 7.9 mM after 16 h, declining thereafter). (b,c) Intermediate profiles in batch and flow mode, respectively. Glycerol accumulated strongly in both formats, exceeding isobutanol titers.

In batch reactions, glucose was fully consumed within 24 h, yielding 13.8 mM isobutanol (13.9% on feed and consumed substrate). Formation peaked at 15.4 mM after 16 h (15.6% on feed; 18.4% on consumed glucose) before declining slightly. In continuous flow, glucose conversion was slower and incomplete, with 5.4 mM remaining after 24 h. Isobutanol titers peaked at 7.9 mM after 16 h (7.9% on feed; 9.2% on consumed glucose) and decreased to 5.8 mM at 24 h, consistent with evaporation losses in the open receiver. In both formats, catalytic activity persisted for ∼24 h, after which product formation plateaued, indicating gradual deactivation of at least one enzyme (Figure 4a). In batch reactions, where all enzymes remain in solution, this behavior is most consistent with gradual inactivation of one or more cascade enzymes. In the continuous‐flow system, however, additional factors may contribute. Because immobilization relies on His‐tag/Ni‐NTA interactions, flow operation may also experience reduced performance from gradual enzyme leaching during extended flow operation [41, 42]. As enzyme leaching was not quantified in the present study, the relative contributions of enzyme inactivation and enzyme loss cannot be distinguished. Nevertheless, both mechanisms would ultimately decrease the availability of active catalytic sites, resulting in lower redox flux through the cascade and reduced isobutanol production at extended reaction times.

#### 2.5.2 Intermediates and Side Products

Analysis of intermediates revealed substantial diversion of carbon away from isobutanol (Figure 4b,c). In batch, glyceraldehyde accumulated to ∼3–4 mM and persisted, glycerate rose to 12.3 mM at 8 h before falling to ∼4 mM at 24 h, and glycerol increased steadily to ∼26.7 mM at 16 h, remaining near 25 mM thereafter. In flow, glyceraldehyde spiked even higher (6.3 mM at 4 h) but was mostly depleted by 24 h, glycerate remained low (3–5 mM), and glycerol accumulated more strongly, reaching ∼29 mM at 24 h. At this time point, glycerol exceeded isobutanol titers by ∼two‐fold in batch and ∼five‐fold in flow, representing the major pathway diverting carbon from the target isobutanol product and thereby limiting overall cascade efficiency. When considering both glycerol and isobutanol as products, the efficiency for an initial setup and proof of concept of the immobilization strategy is not as bad with ca. 42 and 38 mM of products formed for batch and flow, respectively.

#### 2.5.3 Mechanistic Basis for Glycerol Diversion

The observed glycerol accumulation likely reflects the reported substrate preference of free terminal *Tk*ADH toward glyceraldehyde. Kinetic characterization showed a *V*_max_ of 4.5 U mg^−1^ and *K*_m_ of 3.6 mM for glyceraldehyde, versus a higher *V*_max_ of 8.2 U mg^−1^ but larger *K*_m_ of 12.5 mM for isobutyraldehyde (Figure S12). At the subsaturating substrate concentrations in the cascade, this lower *K*_m_ confers nearly two‐fold higher catalytic efficiency toward glyceraldehyde. Combined with continuous glyceraldehyde supply from the KDG‐KDGA split (Figure 1), this kinetic bias is likely to direct reducing equivalents toward glycerol at the expense of isobutanol.

Adding to this, one can consider that with the fast reaction of GDH, ADH will have a fast delivery of the reduced cofactor. On the other hand, ALDH is relying on the likewise slow KARI enzyme to regenerate NAD^+^. In this constellation, it seems not surprising that the competition for glyceraldehyde as a substrate is partially won by the ADH. Unfortunately, while there are more specific ADH available, also at our hands, the necessity for a monomeric enzyme forced us to not utilize these here.

#### 2.5.4 Factors Limiting Overall Performance

Several additional factors contributed to the modest isobutanol titers relative to cascades with free cofactors. Enzyme loading ratios were not systematically reoptimized after introducing fusion constructs, leading to mismatched activity between fast upstream and slower downstream reactions. The kinetic bias of *Tk*ADH toward glyceraldehyde, coupled with the intrinsic volatility of isobutyraldehyde, further limits conversion: isobutyraldehyde can be diverted competitively or lost via evaporation before reduction to isobutanol. In addition to enzyme inactivation, gradual enzyme leaching from the immobilized reactor may contribute to the decline in activity during continuous operation and represent an important target for future process optimization. Process‐level losses, including evaporation of isobutanol in the open flow receiver, also reduce apparent yields, as a fraction partitions into the gas phase before analysis. Despite these limitations, nanomachine fusions sustain redox flux in both batch and continuous‐flow formats, enabling direct glucose‐to‐isobutanol conversion without free cofactors. The data highlight several levers for improvement: optimizing enzyme loadings, replacing the terminal ADH with variants exhibiting higher selectivity for isobutyraldehyde, protecting the tethered cofactors to extend their turnover number (e.g., by reducing hydrolysis or oxidative degradation of the cofactor–linker conjugate), and operating in closed‐flow systems to minimize evaporation. Addressing these constraints will be essential to approach free‐cofactor performance, and the study establishes a quantitative benchmark for future optimization.

### 2.6 Quantifying Cofactor Durability in Tethered Nanomachines

**FIGURE 5:**
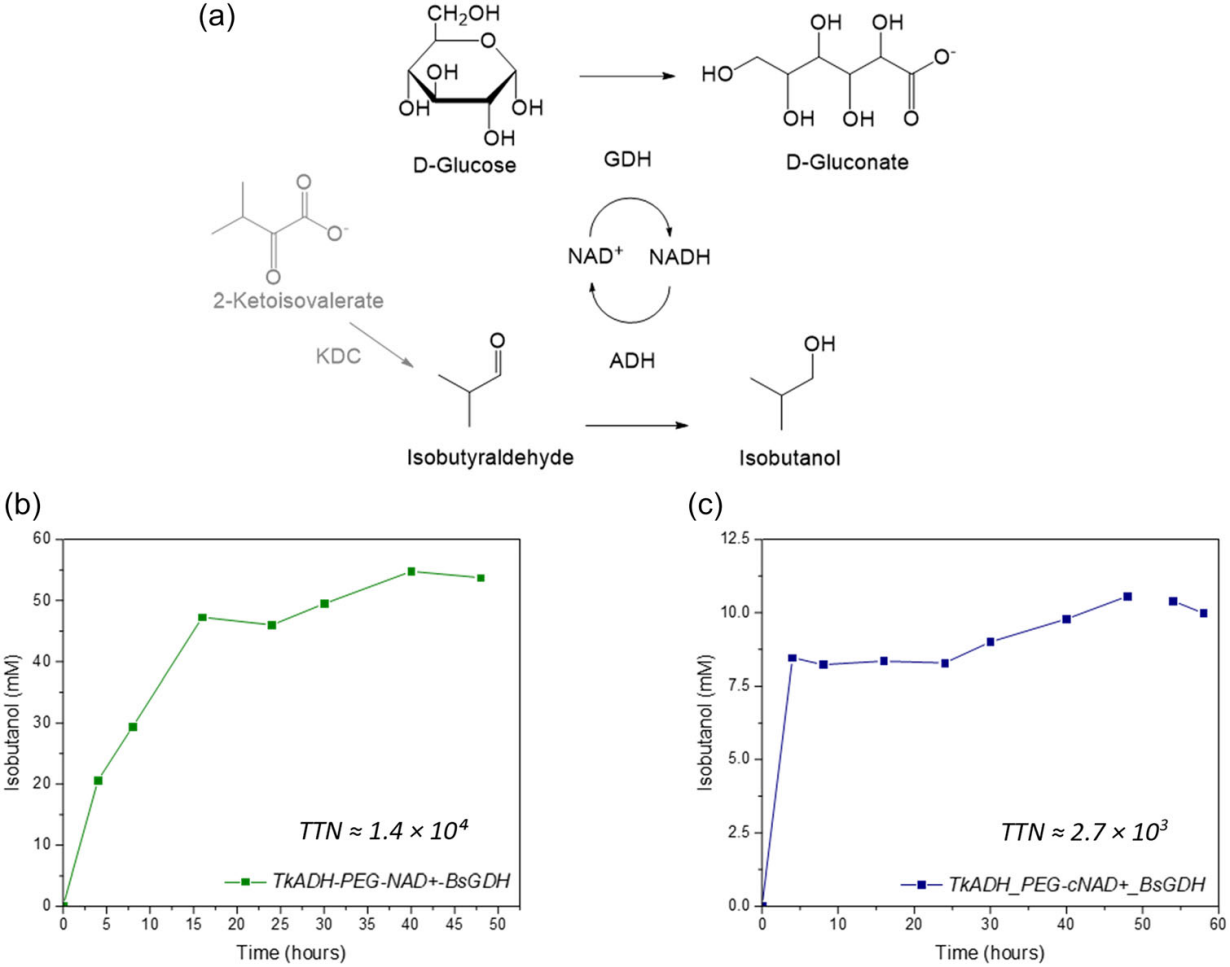
Total turnover number (TTN) analysis of tethered redox nanomachines. (a) Schematic of the minimized reaction system used to quantify cofactor turnover. The GDH domain oxidizes glucose to regenerate NADH, which is utilized by the ADH domain to reduce isobutyraldehyde supplied in situ via KDC‐mediated decarboxylation of ketoisovalerate. (b) Time course of isobutanol production catalyzed by the ADH–GDH nanomachine with tethered PEG–NAD^+^. Endpoint values were used to calculate a TTN of ∼1.4 × 10^4^ per cofactor molecule. Control reactions lacking either KDC or the nanomachine showed no detectable isobutanol formation by HPLC and are omitted for clarity. (c) TTN assay of the same nanomachine carrying PEG–cNAD^+^. Product accumulation reached a TTN (∼2.7 × 10^3^), addition of 2 mM free cNAD^+^ after 48 h did not restore activity, indicating that enzyme deactivation or aldehyde limitation, rather than cofactor depletion, accounted for reaction termination.

To assess cofactor durability, we quantified the TTN of MAL‐PEG24‐NAD^+^ in a minimal ADH–GDH nanomachine (Figure 5a). In this system, the GDH domain continuously regenerates NADH from glucose, while ADH reduces isobutyraldehyde to isobutanol. To avoid inhibition by high aldehyde concentrations, isobutyraldehyde was supplied in situ from ketoisovalerate using a rate‐determining small amount of KDC, maintaining a low steady‐state aldehyde pool while sustaining flux. Batch reactions were monitored for 48 h by HPLC, and TTN was calculated as the cumulative moles of isobutanol formed per mole of tethered NAD^+^. From 55 mM isobutanol produced with ∼4 µM enzyme, the TTN was 13,854, indicating each tethered cofactor underwent over 10^4^ cycles before deactivation. These results demonstrate the robustness of PEG‐linked NAD^+^ and confirm its capacity to support extended operation of nanomachines under cell‐free conditions (Figure 5b).

### 2.7 Compatibility of Nanomachines with Synthetic Cofactor cNAD^+^

To evaluate platform modularity, the TTN assay was repeated using MAL‐PEG24‐cNAD^+^, a carba‐NAD^+^ analogue with an ultrastable C—C bond in the nicotinamide riboside. Under identical conditions, the ADH–GDH nanomachine supported isobutanol formation for 48 h, yielding a TTN of ∼2.7 × 10^3^ (Figure 5c). This is lower than the TTN with PEG‐NAD^+^ (∼1.4 × 10^4^), reflecting reduced catalytic efficiency of both ADH and GDH with cNAD^+^ (Figure S14). To determine whether the reaction plateaued due to cofactor depletion or enzyme inactivation, 2 mM free cNAD^+^ was added at 50 h. No further increase in product was observed, indicating that gradual enzyme deactivation or limited aldehyde supply, rather than cofactor shortage, constrained the system. Despite the lower TTN, cNAD^+^ remains attractive for future applications: its C—C bond confers resistance to hydrolytic cleavage and oxidative degradation, providing a longer half‐life than NAD^+^ under elevated temperatures or prolonged operation. With enzyme partners optimized for cNAD^+^, tethered nanomachines could exploit these stability advantages, maintaining activity long after NAD^+^‐based systems would fail.

## 3 Conclusion

Here we demonstrate that the nanomachine concept can be extended beyond individual enzymes to operate an integrated, redox‐dependent multienzyme cascade. By embedding covalently tethered cofactors within fusion enzymes, we enable sustained multistep redox catalysis without the addition of free NAD^+^, and validate this strategy in a glucose‐to‐isobutanol pathway under both batch and continuous‐flow conditions. This addresses a long‐standing limitation in cell‐free biocatalysis, where cofactor supply, retention, and recycling have constrained the deployment of complex redox networks, particularly in flow. The tethered nanomachines remained catalytically competent within the full cascade and supported stable operation for ≈24 h. Importantly, intramolecular cofactor recycling remained effective even when multiple redox steps were combined in a single, noncompartmentalized reaction environment. This establishes that redox‐neutral enzyme networks can be operated in continuous flow using self‐sufficient biocatalysts, rather than relying on external cofactor supplementation or spatial separation of reaction steps.

Overall product yields were lower than those reported for optimized free‐enzyme cascades, and these differences can be rationalized by design trade‐offs intrinsic to the present implementation. Fusion constructs were selected primarily for structural compatibility and reliable cofactor transfer rather than maximal catalytic activity or specificity. For example, *Tk*ADH, despite having a lower *k*_cat_, was chosen over *Bst*ADH because its monomeric architecture facilitated fusion, while attempts to monomerize oligomeric enzymes imposed additional constraints. More generally, enzyme fusion reduces the effective concentration of individual activities relative to freely diffusing enzymes. In addition, whereas earlier implementations exploited elevated temperatures enabled by enzyme thermostability, the present flow configuration was limited to room temperature by column hardware, further reducing catalytic rates.

Despite these trade‐offs, the present work represents a conceptual step change. We show that an eight‐enzyme, 10‐step redox‐neutral cascade can be operated in continuous flow using exclusively tethered cofactors, even when all components are combined in a single reaction mixture. This demonstrates that cofactor self‐sufficiency can be achieved at the level of an integrated network, rather than isolated enzyme pairs, and that intramolecular cofactor transfer remains effective under flow conditions. Beyond natural cofactors, we further establish compatibility of the nanomachine framework with the ultrastable synthetic analogue cNAD^+^. Although the TTN achieved with PEG‐cNAD^+^ was lower than with PEG‐NAD^+^, this limitation arose from reduced enzyme‐cofactor compatibility rather than from cofactor degradation. These results indicate that, once a cofactor is successfully tethered, long‐term performance is governed primarily by enzyme robustness and cofactor compatibility. At the same time, they underscore the modularity of the nanomachine architecture: alternative cofactors can be introduced without altering the tethering strategy, shifting the central optimization challenge toward identifying or engineering enzyme partners with appropriate cofactor tolerance.

The successful implementation of nanomachines within a complex multienzyme cascade also brings into focus the design constraints that currently accompany this technology. Fusion architecture imposes fixed stoichiometries between paired enzymes, limiting the ability to independently tune pathway components. As cascade complexity increases, differences in catalytic efficiency, substrate specificity, and operational stability between fused domains become increasingly important determinants of overall network performance. While these constraints currently limit productivity relative to optimized free‐cofactor systems, they simultaneously identify clear opportunities for further engineering and optimization.

Future improvements will therefore focus on enzyme‐centric optimization, including engineering variants with enhanced activity and specificity towards the substrate as well as altered cofactor, rebalancing enzyme stoichiometries guided by kinetic modeling, and adapting reactor designs to minimize product losses during flow operation. While current performance metrics do not yet match those of free‐cofactor systems, this study establishes a foundation for translating nanomachine‐based cofactor recycling from individual reactions to integrated, multistep redox cascades. More broadly, it defines a viable strategy for constructing self‐sufficient cell‐free biocatalytic networks capable of sustained operation in continuous‐flow formats.

## Funding

This work was supported by Bundesministerium für Bildung und Forschung (Grant 031B1055).

## Conflicts of Interest

The authors declare no conflicts of interest.

## Supporting information

Supplementary Material

## Data Availability

The data that supports the findings of this study are available in the Supporting Information of this article.
