# Supplementary material for: Extending Cofactor‐Tethered Nanomachines to Complex Multienzyme Redox Cascades in Continuous Flow
Source: ChemSusChem. 2026 Jul 24;19(15):e70914. doi: 10.1002/cssc.70914 (PMC13400903; doi:10.1002/cssc.70914)
Supplement: Supplementary file 1 — Supplementary Material [file CSSC-19-e70914-s001.pdf]

Supporting Information

Extending Cofactor-Tethered Nanomachines to Complex Multienzyme Redox Cascades in Continuous Flow

Zinnia Dsouza[a], Jan-Simon Jeshua Friedrichs[a], Okke Melse[a], Gerhard Schenk[b], Volker Sieber* [a], [b]

[a] Z. Dsouza, J.J. Friedrichs, O. Melse, V. Sieber
Chair of Chemistry of Biogenic Resources,
Campus Straubing for Biotechnology and Sustainability, Technical University of Munich
Schulgasse 16, 94315 Straubing, Germany

[b] G. Schenk, V. Sieber
School of Chemistry and Molecular Biosciences
The University of Queensland
68 Copper Road, St. Lucia 4072, Australia

Contents

Experimental Methods 2

Supplementary Tables 18

Supplementary Figures 22

Supplementary References 40

Experimental Methods

Reagents and materials

All chemicals used in this work were obtained with analytical grade from Sigma-Aldrich, Carl Roth, Roche, Serva, or Merck, unless otherwise noted in the respective protocols. Restriction enzymes for cloning and associated cloning reagents were purchased from New England Biolabs. Synthetic genes were ordered from GeneArt (Thermo Fisher Scientific), with codon optimization for *E. coli* expression. Oligonucleotides used as primers for gene amplification as well as custom complementary oligonucleotide strands designed to form the cysteine-containing linker peptide for fusion constructs, were synthesized by Eurofins Genomics. *E. coli* DH5α was used for cloning, and *E. coli* BL21 (DE3) Star (Thermo Fisher Scientific) was used for protein expression.

Fusion Design and cloning

Fusion constructs were designed to encode two sequential oxidoreductases joined by a custom cysteine-containing peptide linker to permit site-specific cofactor tethering. The linker was generated by annealing two complementary synthetic oligonucleotides (Eurofins Genomics), using a thermal protocol of 95 °C for 2 min followed by gradual cooling to room temperature over 45 min.

Genes of interest were either synthesized de novo (GeneArt, Thermo Fisher Scientific) or amplified from existing plasmids. PCR amplification was carried out with Q5 High-Fidelity DNA Polymerase (New England Biolabs), introducing overlaps with both the vector backbone and linker sequence for seamless assembly. The expression vector pET-28a

(+) (N-terminal His_6-tag, kanamycin resistance) was linearized by restriction digestion (NcoI/XhoI) and gel-purified. PCR products, annealed linker, and the digested backbone were combined in Gibson Assembly reactions (New England Biolabs) according to the manufacturer's instructions. The resulting assemblies were transformed into chemically competent *E. coli* DH5α. Colonies were screened by colony PCR, and plasmids from positive clones were purified and verified by Sanger sequencing (Eurofins Genomics).

Expression and purification of enzymes

All enzymes of the multienzymatic cascade, candidate oxidoreductases for creating fusions, and the engineered fusion constructs were expressed and purified using protocols as described here.

Expression: Chemically competent *E. coli* BL21 (DE3) Star cells (Thermo Fisher Scientific) were transformed with 1 µL of sequence-verified plasmid DNA and plated on LB agar containing 50 µg/mL kanamycin. A single colony was inoculated into 25 mL LB-kanamycin (50 µg/mL) and grown overnight at 37 °C, 140 rpm. The pre-culture (10 mL) was used to inoculate 1 L ZYP-5052 auto-induction medium supplemented with kanamycin (50 µg/mL). Cultures were incubated at 37 °C, 110 rpm for 4 h, then shifted to 20 °C, 110 rpm for ~16 h to induce expression. Cells were harvested by centrifugation (5,000 × g, 15 min, 4 °C) and stored at –80 °C until purification.

Purification: Cell pellets were resuspended to a maximum cell concentration of 20% (w/v) in binding buffer (20 mM HEPES, 250 mM NaCl, 20 mM imidazole, 5% glycerol, pH 7.5) (for fusion enzymes, supplemented with protease inhibitors (Roche)), 5 mM $MgCl_2$, and

DNase I (5 µg/mL, AppliChem). Lysis was performed by sonication using an ultrasonicator UIS250 V (Hielscher Ultrasonics) on ice (15 minutes total time; amplitude 70%; 0.5 duty cycle). Insoluble debris was removed by centrifugation (20,000 × g, 30 min, 4 °C), and the clarified lysate was filtered through a 0.45 µm PES membrane.

His-tagged proteins were purified by Ni^{2+}-NTA affinity chromatography using HisTrap™ or GraviTrap™ columns (Cytiva). Bound proteins were eluted with elution buffer (20 mM HEPES, 250 mM NaCl, 500 mM imidazole, 5% glycerol, pH 7.5). Eluates were exchanged into storage buffer (50 mM HEPES, 250 mM NaCl, 5% glycerol, pH 7.5) using PD-10 desalting columns (Cytiva). Protein yields were quantified by UV absorbance at 280 nm (NanoPhotometer®, Implen), and purity was assessed by SDS-PAGE. Proteins were aliquoted, snap-frozen in liquid nitrogen, and stored at –80 °C until use.

Enzyme Activity Assays and Kinetic Measurements

The activities of individual oxidoreductase enzymes, both in their free forms and as domains within fusion constructs, were evaluated spectrophotometrically at 37 °C in 50 mM HEPES buffer (pH 7.5), using NADH formation or consumption as the readout. Assays were performed in 96-well microplates (Greiner Bio-One, polystyrene, F-bottom, clear), and absorbance changes at 340 nm were recorded using an Epoch 2 microplate reader (BioTek Instruments). The total reaction volume in each well was 200 µL. The molar extinction coefficient for NADH ($\varepsilon_{340} = 6.22\ L\ mmol^{-1}\ cm^{-1}$) was used to calculate enzyme activity. All enzyme concentrations were diluted to ensure linearity of the initial rate, and each assay was performed in triplicate. Glucose dehydrogenase (GDH) activity was determined by monitoring NADH formation following the addition of 10 mM D-glucose

and 2.5 mM NAD^+. Alcohol dehydrogenase (ADH) activity was assayed by measuring the decrease in NADH absorbance in the presence of 10 mM isobutyraldehyde and 0.5 mM NADH. Aldehyde dehydrogenase (ALDH) activity was measured by tracking NADH formation using 10 mM D-glyceraldehyde and 2.5 mM NAD^+. Ketol acid reductoisomerase (KARI) activity was quantified using a coupled assay with acetolactate synthase from *Bacillus subtilis* (*Bs*ALS) as the upstream enzyme. The reaction mixture contained 10 mM pyruvate, 5 mM $MgCl_2$, 0.5 mM thiamine pyrophosphate (TPP), 0.5 mM NADH, and *Bs*ALS (5 U/mL) in excess. NADH consumption was monitored at 340 nm as an indirect measure of KARI activity.

For steady-state kinetic analysis, substrate and/or cofactor concentrations were varied while maintaining all other conditions constant. The slope of the initial linear phase of the absorbance-time curve was used to calculate initial reaction rates. Kinetic parameters (K_M, V_{max}) were determined by nonlinear regression fitting to the Michaelis–Menten model using OriginPro 2021b (OriginLab). Turnover numbers (k_{cat}) were calculated based on the enzyme's molar concentration. All results are reported as the mean ± standard deviation of at least three independent measurements.

Analytical size-exclusion chromatography (SEC)

The oligomeric states of the purified free and fused oxidoreductase variants were determined by analytical size-exclusion chromatography (SEC). Chromatographic separation was performed using a Superdex 200 Increase 10/300 GL column (GE Healthcare), operated at a flow rate of 0.5 mL/min and pre-equilibrated with 50 mM potassium phosphate buffer (pH 7.2) containing 150 mM NaCl. The column was calibrated using a gel filtration standard kit (Cytiva), consisting of globular proteins with known

molecular weights. Elution volumes (Ve) for each standard protein were determined and used to calculate the partition coefficient (K_{av}) according to the following equation:

$$K_{av} = \frac{Ve - Vo}{Vc - Vo}$$

where Ve is the elution volume of the protein, Vo is the void volume (determined using Blue Dextran 2000), and Vc is the total column volume. A calibration curve was constructed by plotting K_{av} versus the logarithm (base 10) of the molecular weights of the standards (Figure S6). Molecular weights of the unknown proteins were estimated by interpolation from this standard curve. The quaternary structure of each enzyme variant was assigned based on its estimated molecular weight and compared to its theoretical monomer mass calculated from sequence data. Chromatographic data were acquired and analyzed using UNICORN software (GE Healthcare).

Thermal Stability Analysis by Thermofluor Assay: The thermal stability of free and fused oxidoreductases was evaluated using a Thermofluor assay, which monitors protein unfolding based on the fluorescence of SYPRO Orange dye. Upon gradual heating, the dye binds to hydrophobic regions exposed during thermal denaturation, resulting in an increase in fluorescence signal. Each 30 µL reaction mixture contained 5 µL enzyme solution (1 mg/mL), 5 µL SYPRO Orange dye (62× stock in DMSO; Invitrogen), 5 µL HEPES buffer (pH 7.4), and 15 µL nuclease-free water, resulting in a final HEPES concentration of 50 mM. Samples were loaded into white 96-well PCR plates, sealed with optical adhesive film, and centrifuged briefly to remove bubbles. Fluorescence was recorded using a Bio-Rad CFX96 Real-Time PCR thermal cycler, utilizing the FRET channel (excitation/emission: 470/570 nm). The thermal program included an initial hold

at 4 °C for 5 minutes, followed by stepwise heating from 4 °C to 100 °C at a ramp rate of 0.5 °C every 5 seconds. Melting temperatures (Tm) were determined from the maximum of the first derivative of the fluorescence curve, as analyzed using Bio-Rad CFX Manager 3.1 software.

Thermal Denaturation Analysis by Circular Dichroism (CD) Spectroscopy: Thermal unfolding of fused oxidoreductases was monitored by far-UV CD spectroscopy using a JASCO J-1500 spectropolarimeter. Protein samples were prepared at 20 μM concentration in 10 mM potassium phosphate buffer (pH 7.5). Measurements were conducted in a 0.1 mm path length quartz cuvette. Thermal denaturation was followed by monitoring the CD signal at 220 nm over a temperature range of 24 °C to 94 °C. The temperature was increased at a rate of 1 °C per minute. Melting temperatures (Tm) were determined from the thermal unfolding curves by fitting the data to a Boltzmann sigmoid equation: $y = A_2 + \frac{A_1 - A_2}{1 + e^{\frac{T - T_m}{dT}}}$

where A1 and A2 represent the pre- and post-transition ellipticity baselines, T is the temperature, Tm is the melting temperature, and dT is the slope of the transition. Data fitting and analysis were performed using OriginPro (Version 2021b).

Computational modelling of fusion constructs and peg–NAD$^+$ tethering

Structural models of the fusion constructs were generated using AlphaFold v2.3, yielding high-confidence predictions of domain architecture and linker flexibility. The maleimide-functionalized PEG-NAD$^+$ molecule was designed using ChemDraw and energy-

minimized in Chem3D. The optimized structure was exported in. mol2 format for integration with protein models. To visualize potential PEG-NAD$^+$ conjugation, both the AlphaFold-predicted protein structure and the PEG-NAD$^+$ ligand were imported into Maestro (Schrödinger Suite 2022). Protein preparation involved protonation and the addition of missing heavy atoms using Maestro's Protein Preparation Wizard. The PEG-NAD$^+$ molecule was manually positioned near the cysteine residue located within the flexible GSS linker, and a covalent bond was formed between the maleimide moiety and the thiol group of cysteine. The resulting structure was energy minimized locally and exported as a PDB file for structural inspection and linker geometry analysis. To investigate the dynamic conformational flexibility of the fusion construct and evaluate potential reach of tethered cofactors, molecular dynamics (MD) simulations were performed using the apo structure of fusion constructs. System setup and simulation setup followed established protocols,[1] with the following modifications: a) The ff19SB force field was used instead of ff14SB, to better model large domain dynamics and flexible linkers. b) The system was solvated using OPC water instead of the commonly used TIP3P model, as OPC provides improved accuracy in combination with ff19SB. c) A 4 femtosecond time step was used in production runs. To enable this, hydrogen mass repartitioning was applied using the 'HMassRepartition' command in ParmEd, redistributing a portion of the heavy atom mass to bonded hydrogen atoms (Amber documentation: Amber22 Manual). The system (fusion protein) was protonated, solvated, neutralized, energy-minimized, and gradually heated prior to production runs. The simulation data was analyzed to evaluate interdomain flexibility and linker extension behavior, informing the design of suitable PEG linker lengths for subsequent covalent tethering.

Synthesis of PEG-modified nicotinamide cofactors

Materials Maleimide-PEG24-NHS ester (CAS 756525-92-5) was obtained from Iris Biotech GmbH. Aminopropyl-modified silica resin (Polygoprep 60-30 NH_2, REF 711014.100) was purchased from Macherey-Nagel.

1) Maleimide-PEG_{24}-N6-(2-aminoethyl) -NAD^+

Synthesis of N6-(2-aminoethyl)-NAD^+

The synthesis was adapted from published procedures[2, 3]. In a 100 mL three-necked flask, 10.0 g NAD^+ (1.00 eq., 15.1 mmol) were suspended in 30 mL deionized water. The pH was set to 3.3 using 70% HClO4. While maintaining a pH between 3.3 and 3.5 using 70% HClO4, 2.04 mL aziridine (2.80 eq., 42.3 mmol) were slowly added at 30°C. after the addition of aziridine was completed, the mixture was stirred at 30°C for 48 h. The reaction mixture was then precipitated in ice-cold EtOH and filtered. The residue was dissolved in water and applied to a DOWEX 1x8 ion-exchange resin (formate-form) to remove unreacted NAD^+. The column was flushed with water and the product containing fractions (TLC) collected and lyophilized yielding around 3.71 g crude material. The crude product mainly contained N1-(2-aminoethyl)-NAD^+ and was used for the next reaction step without further purification.

For the Dimroth rearrangement, 840 mg of the crude material were dissolved in 30 mL deionized water. The pH was set to 6.2 - 6.5 with 1 M LiOH and the mixture stirred for 4 h at 50°C. The conversion was monitored by TLC using a solvent mixture consisting of propionic acid, water and 32% ammonia, in a ratio of 6.6/3.3/0.25. After completion of the reaction, the reaction mixture was lyophilized. The crude material was suspended in 400

mL 80% acetonitrile containing 25% of a 0.1% formic acid solution in water (the crude material does not fully dissolve). The mixture was applied to an aminopropyl modified silica column (15.0 x 3.00 cm) equilibrated with 80% acetonitrile (20% 0.1% formic acid). The column was properly flushed with 80% acetonitrile, followed by the elution of the main side-product 2-ethanoadenine-NAD+ between 70% – 65% acetonitrile. By further increasing the content of 0.1% formic acid from 35% – 30%, N6-(2-aminoethyl)-NAD^+ is eluted. The product containing fractions are combined and the acetonitrile removed under reduced pressure at ambient temperature (SpeedVac). The concentrated fractions are lyophilized. In total 105 mg of pure N6-(2-aminoethyl)-NAD^+ were obtained as white powder.

Characterization:

^1H NMR (400 MHz, D_2O) δ 9.39 (d, *J* = 1.6 Hz, 1H), 9.33 – 9.21 (m, 1H), 8.89 (dt, *J* = 8.1, 1.6 Hz, 1H), 8.41 (s, 1H), 8.26 (dd, *J* = 8.1, 6.2 Hz, 1H), 8.20 (s, 1H), 6.13 (d, *J* = 5.4 Hz, 1H), 6.03 (d, *J* = 5.9 Hz, 1H), 4.73 (t, *J* = 5.5 Hz, 1H), 4.61 – 4.52 (m, 2H), 4.53 – 4.48 (m, 1H), 4.48 – 4.44 (m, 1H), 4.43 – 4.34 (m, 2H), 4.32 – 4.16 (m, 3H), 3.91 – 3.84 (m, 2H), 3.33 (t, *J* = 5.7 Hz, 2H).

^{13}C NMR (101 MHz, D_2O) δ 165.41, 154.49, 152.70, 145.87, 142.53, 140.01, 139.64, 133.76, 128.72, 118.91, 100.03, 87.15 (d, *J* = 8.3 Hz), 86.80, 83.89 (d, *J* = 8.6 Hz), 77.64, 74.22, 72.12, 70.77, 70.38, 65.34 (d, *J* = 4.7 Hz), 64.97 (d, *J* = 4.7 Hz), 62.54, 39.33.

ESI-MS: $[M+H]^+$ *m/z* 707.1

Synthesis of Mal-PEG$_{24}$-N6-(2-aminoethyl) --NAD$^+$

The conjugate was synthesized using the published protocol [4]. In a 5 mL round-bottom flask, 100 mg N6-(2-aminoethyl)-NAD+ (1.00 eq., 0.142 mmol) and 247 mg Mal-PEG24-NHS (1.25 eq., 0.177 mmol) were dissolved in 5 mL 10 mM PBS buffer (pH 7.4). The reaction mixture was stirred at ambient temperature for 4 h. The crude reaction mixture was purified using an aminopropyl-modified silica resin. For elution of the product a solvent mixture consisting of 80% water (0.1% formic acid) and 20% acetonitrile was used. Before elution of the product the column was flushed with a lower content of the aqueous phase. The product containing fractions, were combined and lyophilized. The product was obtained as transparent and colourless foam with a yield of 41% (117 mg, 0.0587 mmol).

Characterization:

^{1}H NMR (400 MHz, D_2O) δ 9.30 (s, 1H), 9.15 (d, J = 6.3 Hz, 1H), 8.80 (d, J = 8.0 Hz, 1H), 8.44 (s, 1H), 8.26 (t, J = 1.8 Hz, 1H), 8.16 (s, 1H), 6.75 (s, 1H), 6.21 – 5.93 (m, 2H), 4.50 – 4.34 (m, 4H), 4.28 (s, 2H), 4.14 (d, J = 15.6 Hz, 3H), 3.76 – 3.36 (m, 111H), 3.21 (t, J = 5.3 Hz, 2H), 2.48 – 2.26 (m, 4H).

ESI-MS: $[M+H]^+$ *m/z* 1986.7, $[M+2H]^{2+}$ *m/z* 993.5, $[M+3H]^{3+}$ *m/z* 662.8

2) Maleimide-PEG$_{24}$-N6-(2-aminoethyl) -CarbaNAD$^+$

Carba-NAD$^+$ (cNAD$^+$) was prepared according to previously published procedures[5]. The synthesis of the PEG-functionalized analogue closely followed the protocol used for PEG–NAD$^+$, with few modifications.

Synthesis of N6-(2-aminoethyl)-CarbaNAD⁺

In a 100 mL three-necked flask, 1.28 g CarbaNAD$^+$ (1.00 eq., 1.94 mmol) were suspended in 30 mL deionized water. The pH was set to 3.5 using 70% HClO4. While maintaining a pH between 3.5 and 3.8 using 70% HClO4, 0.402 mL aziridine (4.00 eq., 7.74 mmol) were slowly added at 30°C.After the addition of aziridine was completed, the mixture was stirred at 30°C for 24 h. The reaction mixture was then precipitated in ice-cold EtOH and filtered. The residue was dissolved in water and applied to a DOWEX 1x8 ion-exchange resin (formate-form) to remove unreacted CarbaNAD$^+$. The column was flushed with water and the product containing fractions (TLC) collected and lyophilized yielding around 233 mg crude material. Unreacted CarbaNAD$^+$ was recovered from the column (767 mg, 1.16 mmol) by elution with 0.03 M formic acid. The crude product mainly contained N1-(2-aminoethyl)-CarbaNAD$^+$ and was used for the next reaction step without further purification.

For the Dimroth rearrangement, 230 mg of the crude material were dissolved in 30 mL deionized water. The pH was set to 6.2 – 6.5 with 1 M LiOH and the mixture stirred for 6 h at 50°C. The conversion was monitored by TLC using a solvent mixture consisting of propionic acid, water and 32% ammonia, in a ratio of 6.6/3.3/0.25. After completion of the reaction, the reaction mixture was lyophilized. The crude material was suspended in 400 mL 80% acetonitrile containing 20% of a 0.1% formic acid solution in water (the crude material does not fully dissolve). The mixture was applied to an aminopropyl modified silica column (15.0 x 3.00 cm) equilibrated with 80% acetonitrile (20% 0.1% formic acid). The column was properly flushed with 80% acetonitrile, followed by the elution of the main side-product 2-ethanoadenine-CarbaNAD+ between 70% – 65% acetonitrile. By further

increasing the content of 0.1% formic acid from 35% – 30%, N6-(2-aminoethyl)-CarbaNAD$^+$ is eluted. The product containing fractions are combined and the acetonitrile removed under reduced pressure at ambient temperature (SpeedVac). The concentrated fractions are lyophilized. In total 72.1 mg of N6-(2-aminoethyl)-CarbaNAD$^+$ were obtained as white powder.

Characterization:

ESI-MS: $[M+H]^+$ *m/z* 705.3

Synthesis of Mal-PEG$_{24}$-N6-(2-aminoethyl) - CarbaNAD$^+$

In a 5 mL round-bottom flask, 42.0 mg N6-(2-aminoethyl)-NAD+ (1.00 eq., 0.0833 mmol) and 116 mg Mal-PEG24-NHS (1.00 eq., 0.0831 mmol) were dissolved in 5 mL 10 mM PBS buffer (pH 7.4). The reaction mixture was stirred at ambient temperature for 4 h. The crude reaction mixture was purified using an aminopropyl-modified silica resin. For elution of the product a solvent mixture consisting of 80% water (0.1% formic acid) and 20% acetonitrile was used. Before elution of the product the column was flushed with a lower content of the aqueous phase. The product containing fractions, were combined and lyophilized. The product was obtained as transparent and colourless foam with a yield of 28% (36,7 mg, 0.0233 mmol).

ESI-MS: $[M+2H]^{2+}$ *m/z* 992.5, $[M+3H]^{3+}$ *m/z* 662.2

HPLC-MS analysis:

The HPLC system (Ultimate 3000RS, Dionex) was composed of a degasser (SRD 3400), a pump module (HPG 3400RS), an autosampler (WPS 3000TRS), a column compartment (TCC 3000RS), a diode array detector (DAD 3000RS) and an ESI-ion-trap unit (HCT, Bruker). Data was collected and analyzed with Bruker Hystar, and Dionex Chromelion software. The column (Triat Hilic-Diol, 100 mm length, 2 mm i.d.; 1.9 µm particle size; YMC) was tempered to 7 °C. Mobile phase A consisted of 5 mM ammonium formate buffer (pH 4.5) and mobile phase B consisted of acetonitrile containing 0.1% v/v formic acid. The chromatographic flow rate was set to 0.4 mL/min. The gradient was programmed as following: start of mobile phase A at 15% for 2 min, with increase to 21% over 2 min, hold for 3 min, with following increase to 35% over 2 min. The gradient was hold for 3 min and returned to starting conditions. The starting conditions of mobile phase A at 15% were held for 5 min. Before entering ESI-MS the flow was split 1:20 (Accurate-Post-Column-Splitter, Dionex). Temperature of the autosampler was set to 10 °C and an injection volume of 10 µL was used.

The ion-trap was operated in the ultra-scan mode (26,000 *m*/*z*/s) from 50 to 1500 *m*/*z*. The ICC target was set to 200,000 with a maximum accumulation time of 50 ms and four averages. The ion source parameters were set as follows: capillary voltage 4 kV, dry temperature 325 °C, nebulizer pressure 40 psi and dry gas flow 6 L/min. Auto-MS-mode with the smart target mass of 600 *m*/*z* and a MS/MS fragmentation amplitude of 0.5 V was used. The quantification was performed by using the extracted ion chromatograms (EIC) of the *m*/*z* value corresponding to the protonated molecules.

Cofactor Conjugation and validation

Purified fusion enzymes (20–50 µM) were incubated with a 10-fold molar excess of PEG-NAD^+ in 50 mM potassium phosphate buffer (pH 7.5) at 4 °C with gentle agitation at 350 rpm for approximately 15 hours. To remove excess or unbound cofactor, the reaction mixtures were washed three times with the same buffer using 10 kDa centrifugal filters equipped with modified PES membranes (VWR).

Cofactor conjugation was confirmed using two complementary approaches. 1. Quantification of free thiols. Accessible cysteine residues were quantified using the DTNB (5,5′-dithiobis-(2-nitrobenzoic acid)) assay. Calibration curves were generated from L-cysteine standards (10–100 µM). Assays (250 µL) contained 0.2 mM DTNB in 50 mM potassium phosphate buffer (pH 7.5) and either L-cysteine standard or enzyme sample (~20 µM). Samples were incubated for 20 min at room temperature in the dark, and absorbance was measured at 412 nm.

2. Functional activity in the absence of free NAD^+. Fusion enzymes (~20 µM) were assayed for glucose dehydrogenase (GDH) or aldehyde dehydrogenase (ALDH) activity in 200 µL reactions at 37 °C. Reactions contained 50 mM potassium phosphate buffer (pH 7.5) and 10 mM of the respective substrate. NADH formation was monitored at 340 nm using an Agilent Cary UV–Vis spectrophotometer and optical cuvettes designed for low-volume measurements (ROTILABO®, 10 mm × 2 mm, liquid-tight stopper, glass).

Assembly of multienzyme cascade in batch

Fusion enzymes covalently conjugated with PEG-NAD^+, along with all additional enzymes required for the glucose-to-isobutanol cascade (depicted in Figure 1), were expressed and

purified as described above. Proteins were concentrated using 10 kDa centrifugal filters (VWR). Batch reactions (1 mL total volume) were prepared with enzyme concentrations given in Table S4. The reaction mixture contained 100 mM D-glucose, 100 mM HEPES buffer (pH 7.5), 5 mM $MgCl_2$ and 0.5 mM thiamine pyrophosphate (TPP). Reactions were incubated at room temperature (~22–25 °C) for 24 hours under static conditions. Samples were withdrawn at defined intervals for analysis of glucose consumption and isobutanol formation.

Continuous-flow cascade operation

For continuous flow experiments, the same set of enzymes were immobilized onto a 1 mL HisTrap column (Cytiva; 0.7 × 2.5 cm; ~40 mg binding capacity) via their N-terminal His-tags. The column was equilibrated with 100 mM HEPES buffer (pH 7.5) before loading the enzyme mixture. Immobilization efficiency was verified by monitoring absorbance at 280 nm in the flow-through. The reaction mixture, identical in composition to the batch setup, was continuously recirculated through the enzyme-packed column using a peristaltic pump (GE Healthcare) at a flow rate of 0.8 mL/min (setup shown in Figure S11). Continuous operation was maintained for 24 hours at room temperature, with samples collected at defined intervals for isobutanol quantification.

Minimized coupled-reaction for TTN determination

To determine the total turnover number (TTN) of tethered cofactors, a reduced coupled reaction consisting of Glucose oxidation by the GDH domain and coupled to isobutyraldehyde reduction by the ADH domain was used. Cofactors were covalently

tethered as PEG–NAD^+ or PEG–$cNAD^+$ to this fusion. To prevent aldehyde inhibition, isobutyraldehyde was generated in situ from 2,3-ketoisovalerate using keto-acid decarboxylase (KDC). Reactions (1 mL) contained 100 mM glucose, 100 mM HEPES (pH 7.5), 5 mM $MgCl_2$, 0.5 mM TPP, the nanomachine (*Tk*ADH–$PEG_{24}NAD^+$–*Bs*GDH or *Tk*ADH–$PEG_{24}cNAD^+$–*Bs*GDH), 2,3-ketoisovalerate (100 mM) and (0.15 mg/mL) KDC. Incubations were performed at room temperature for up to 48 h, with time-course samples analyzed for isobutanol. TTNs were calculated as moles of isobutanol formed per mole of tethered cofactor.

Isobutanol analysis by HPLC

Reaction samples were diluted 10–30-fold in 2.5 mM H_2SO_4 and filtered through 10 kDa centrifugal filters (VWR) to remove proteins. Isobutanol and intermediates were quantified using an Ultimate 3000 HPLC system equipped with an autosampler, a diode-array detector (Dionex Softron, Germering, Germany), and a refractive index detector (RI-101, Shodex, Gersthofen, Germany). Separation was achieved on a Rezex ROA-Organic Acid H^+ (8%) column (Phenomenex) under isocratic elution with 2.5 mM H_2SO_4 at 70 °C for 42 min. The HPLC-detected compounds (D-glucose, D-glyceraldehyde, sodium glycerate, glycerol, and isobutanol) are shown in Figure S13. Peak assignment and quantification were performed using Chromeleon Software 6.8 (Thermo Fisher Scientific).

Supplementary Tables

Table S1: Kinetic parameters of oxidoreductase variants from the glucose-to-isobutanol cascade. Steady-state kinetic data (V_{max}, K_m and k_{cat} values) were determined for alternative GDH, ADH, ALDH and KARI candidates under standard assay conditions as specified in the Methods section. These values were used to guide the selection of enzyme variants for the construction of biocatalytic nanomachines.

**Enzyme**	**Organism**	**V_{max} (U/mg)**	**k_{cat} (s^{-1})**	**K_M (mM) Substrate**	**K_M (mM) Cofactor**
Glucose Dehydrogenase (GDH)					
*Bs*GDH	*Bacillus subtilis*	173	87	3.0	0.90
*Ss*GDH	*Sulfolobus solfataricus*	10	7.1	1.9	0.20
Alcohol Dehydrogenase (ADH)					
*Bs*ADH	*Geobacillus stearothermophilus*	72	46	2.7	0.14
Aldehyde Dehydrogenase (ALDH)					
*Ta*ALDH (M42)	*Thermoplasma acidophilum*	2.7	2.6	1.3	1.5
*Hs*AlDH	*Herbaspirillum seropedicae* Z67 (variant)	78	69	3.1	0.40
Ketol Acid Reductoisomerase (KARI)					
*Mr*KARI	*Meiothermus ruber*	1.5	1.0	1.4	0.09

Table S2: *In silico* analysis of oxidoreductase candidates for fusion design. Predicted structural features of enzyme variants were evaluated to assess their suitability for nanomachine construction. The analysis included overall fold, orientation of catalytic sites and the accessibility of the N- and C-termini to guide linker placement and fusion strategy.

Enzyme	PDB ID	Seq. Id	Ions	N-Termini	C-Termini	Notes/Implications
*Bs*GDH	1RWB	83.5%		Short helix; tolerable for fusion	Long structured loop; not optimal	N-terminus preferred
*Ss*GDH	2CDA	100%		Within β-strand; poor choice	Within β-strand; slightly better than N-term	Neither ideal
*Bst*ADH	1RJW	100%	Zn_2^+	Within β-strand; not optimal	Loop after β-strand; suitable	C-terminus favourable
*Ta*ALDH (M42)	5J77<br>5M4X	98.8%<br>99.0%		Long loop; accessible	Small helix after loop; may affect dimerization	N-terminus favourable
*Hs*ALDH	5X5T	65.1%		Long loop; accessible	Ends in β-strand; poor	N-terminus favourable
*Mr*KARI	4TSK<br>1NP3<br>4XIY<br>6AQJ	64.6%<br>62.2%<br>61.6%<br>59.6%	Mg_2^+	Flexible loop; likely usable	Long loop ending in helix; inconsistently resolved	Both termini acceptable, N-term preferred

Table S3: Overview of oxidoreductases analysed for fusion construct design. The quaternary structure and the number of cysteine residues (suitability for site-specific cofactor tethering) were evaluated as key factors.

No.	Enzyme	Quaternary State	No. of Cysteine
1	*Ss*GDH	Tetramer	6
2	*Bs*GDH	Tetramer	0
3	*Bst*ADH	Tetramer	9
4	*Tk*ADH	Monomer	2 (Disulfide bridge)
5	*Ta*ALDH	Trimer	2
6	*Hs*ALDH	Tetramer	2 (Catalytic cysteines)
7	*Mr*KARI	Dodecamer	2
8	*Ia*KARI	Monomer	1

Multienzyme cascade: Transformation of D-Glucose to Isobutanol

Table S4: Final concentrations (mg/mL) of cascade enzymes used in the glucose-to-isobutanol pathway under batch and continuous-flow conditions. Values for free enzymes were adapted from previous modeling studies, while fusion-enzyme nanomachines were included at the indicated concentrations.

Enzyme	Source	Enzyme Function	Substrate	Final Concentration (mg/mL)
Free enzymes				
*Pu*DHT	*Paralcaligenes ureilyticus*	Dihydroxy acid dehydratase	Gluconate	6.92
*Ft*DHAD	*Fontimonas thermophila*	Dihydroxy acid dehydratase	Glycerate, Dihydroxyisovalerate	2.28
*Pt*KDGA	*Picrophilus torridus*	KDG aldolase	2-keto-3-deoxygluconate	0.59
*Bs*ALS	*Bacillus subtilis*	Acetolactate synthase	Pyruvate	0.74
KDC (7 M.D)	*Lactococcus lactis (variant 7M.D)*	Ketoacid decarboxylase	2-ketoisovalerate	0.15
Nanomachines				
*Tk*ADH-PEG$_{24}$NAD$^+$-*Bs*GDH				0.5
*Ia*KARI-PEG$_{24}$NAD$^+$-*Hs*ALDH				4.5

Supplementary Figures

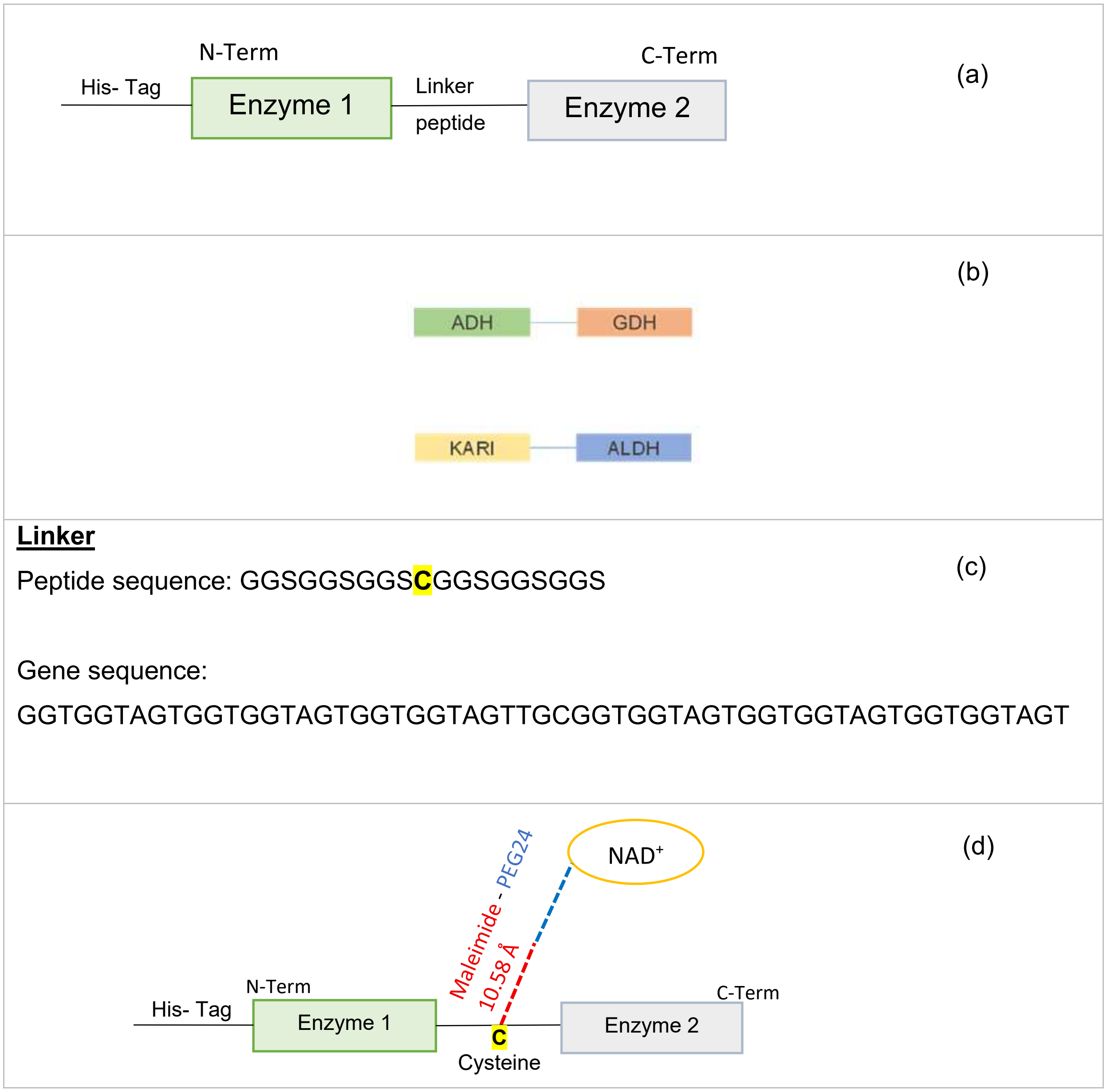

Figure S1: Design of fusion constructs. (a) Schematic representation of a generic fusion construct showing the N-terminal His6-tag, two oxidoreductase domains, connected by a Gly–Ser peptide linker. (b) Specific fusion pairs selected for this study: a glucose dehydrogenase (GDH) fused to an alcohol dehydrogenase (ADH), and a ketol acid reductoisomerase (KARI) fused to an aldehyde dehydrogenase (ALDH). (c) Nucleotide and amino acid sequences of the customised linker, highlighting the central cysteine residue used for site-specific conjugation of PEG–NAD^+. (d) Schematic of the fusion construct highlighting the engineered cysteine within the Gly–Ser linker

used for site-specific conjugation of maleimide–PEG–NAD^+; the cysteine–PEG attachment distance is ~10.6 Å.

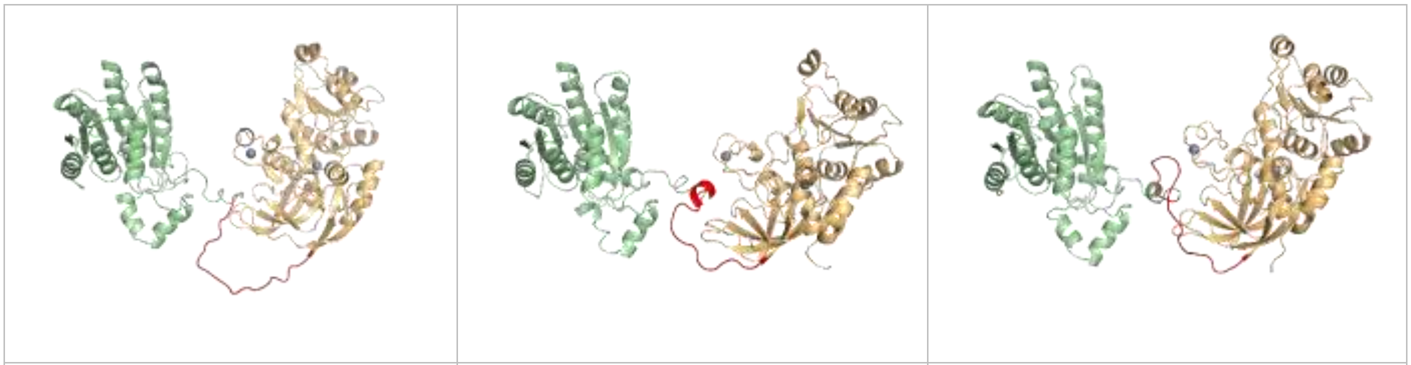

0 ns	90 ns	180 ns

Figure S2: Molecular dynamics simulations reveal interdomain flexibility of the apo fusion construct. Representative trajectory snapshots show that the flexible linker (red) permits largely independent motion of the two enzyme domains (green and gold) without compromising domain structural integrity.

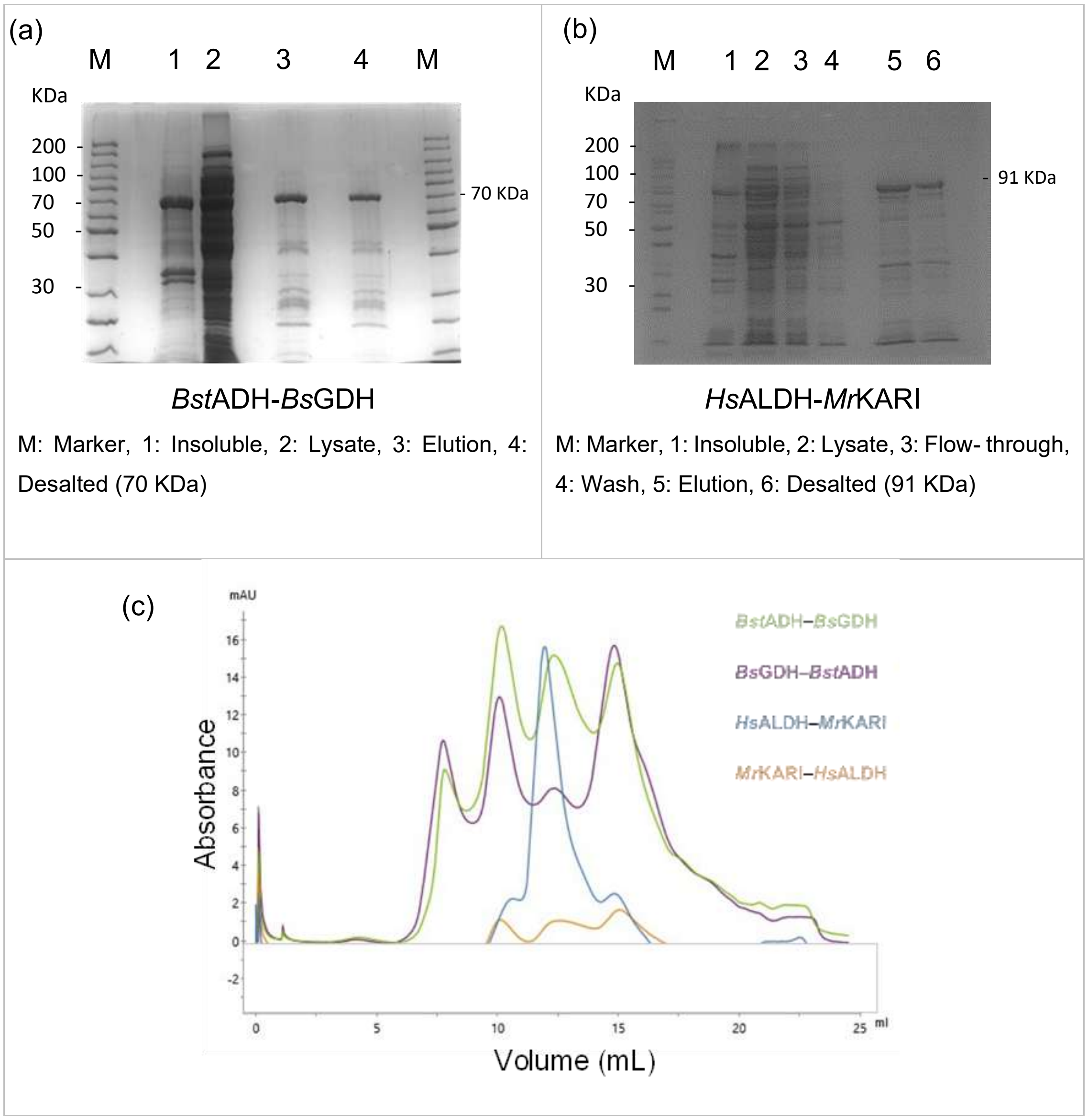

Figure S3: Expression profiles and oligomerization of early fusion designs. (a) SDS–PAGE analysis of the *Bst*ADH-*Bs*GDH fusion construct (MW ~70 kDa). The detectable band corresponds to highly concentrated fractions, showing poor soluble expression. (b) SDS–PAGE analysis of the HsALDH–MrKARI concentrated fusion proteins (MW ~91 kDa), likewise exhibiting low solubility. (c) Size-exclusion chromatography (SEC) profiles of all initial fusions overlaid, revealing multiple elution peaks consistent with heterogeneous high-order assemblies and misfolding.

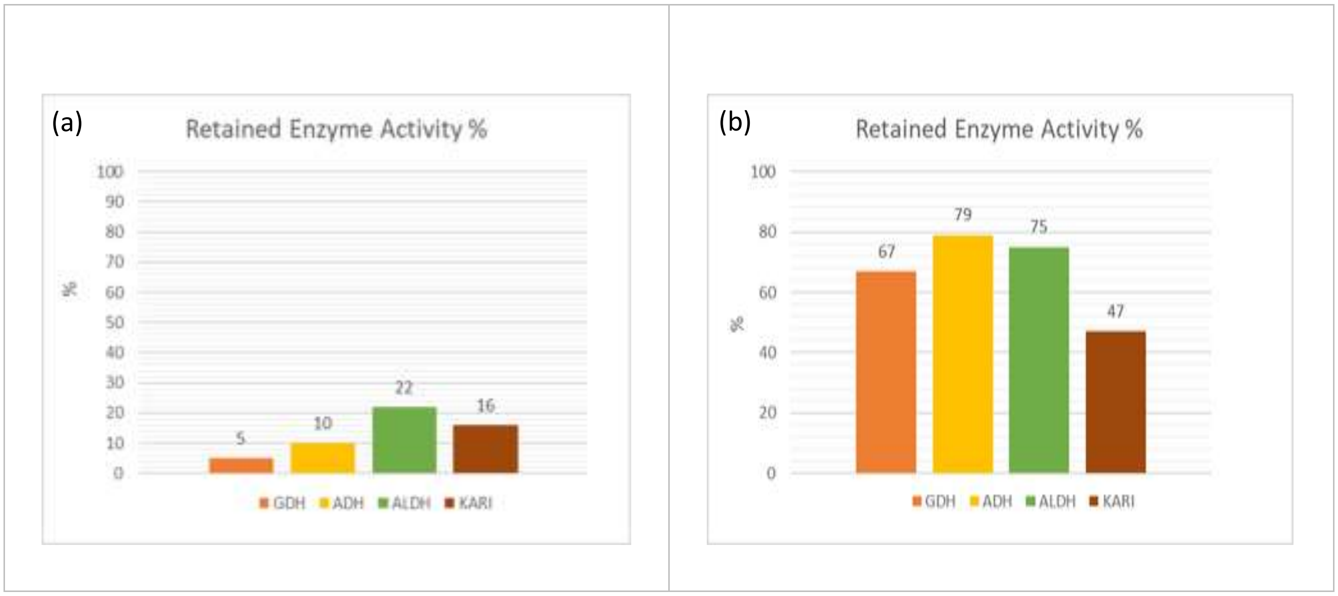

Figure S4: Activity retention in fusion constructs before and after redesign. (a) Relative enzyme activities of each enzyme in initial fusion constructs compared to their corresponding free enzyme counterparts. In the construct the enzymes show sharply reduced activity, with losses ranging from 78% (ALDH) to 95% (GDH). (b) In the redesigned fusion constructs, in which two oligomeric enzymes were replaced by monomeric variants, a significant improvement of retained catalytic activity is observed (as high as 79% - for ADH). The relative improvements vary from ~three-fold (for KARI, from 16% to 47%) to over 13-fold (for GDH, from 5% to 67%). Activities were measured under standard assay conditions and normalized to the activity of the corresponding free enzyme (set to 100%).

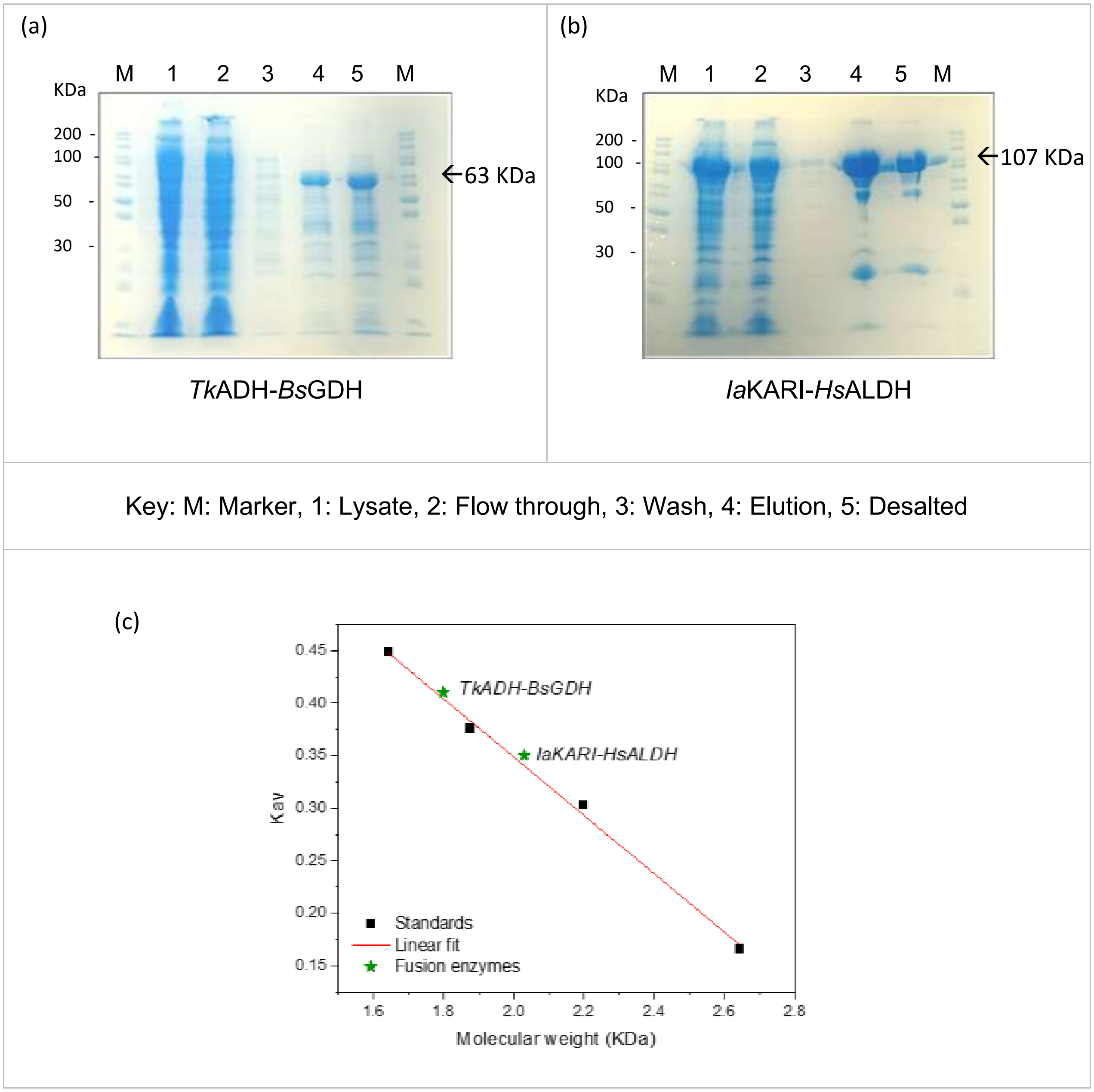
(a)
M 1 2 3 4 5 M
KDa
200
100
50
30
←63 KDa
TkADH-BsGDH
(b)
M 1 2 3 4 5 M
KDa
200
100
50
30
←107 KDa
IaKARI-HsALDH
Key: M: Marker, 1: Lysate, 2: Flow through, 3: Wash, 4: Elution, 5: Desalted
(c)
Kav
0.45
0.40
0.35
0.30
0.25
0.20
0.15
TkADH-BsGDH
IaKARI-HsALDH
Standards
Linear fit
Fusion enzymes
1.6
1.8
2.0
2.2
2.4
2.6
2.8
Molecular weight (KDa)

(d)

Protein	Ve (mL)	K_{av}	MW Monomer (KDa)	Calculated (MW)	Quaternary state
Ovalbumin	14.9	0.45	44	44	Standards
Conalbumin	13.7	0.38	75	80	
Aldolase	12.5	0.30	158	146	
Ferritin	10.2	0.17	440	454	
*Tk*ADH-*Bs*GDH	14.30	0.41	63	59	Monomer
*Ia*KARI-*Hs*ALDH	13.20	0.35	107	102	Monomer

Figure S5: Expression profiles and oligomeric states of redesigned fusion constructs. (a) & (b) SDS–PAGE analysis showing enhanced expression of the redesigned fusion constructs (*Tk*ADH–*Bs*GDH and *Ia*KARI–*Hs*ALDH, respectively) when compared to the original systems (see **Figure S3**). (c) & (d) Size-exclusion chromatography and calibration analysis confirming that the redesigned fusions elute as monomeric species, with their apparent molecular weights consistent with the calculated values.

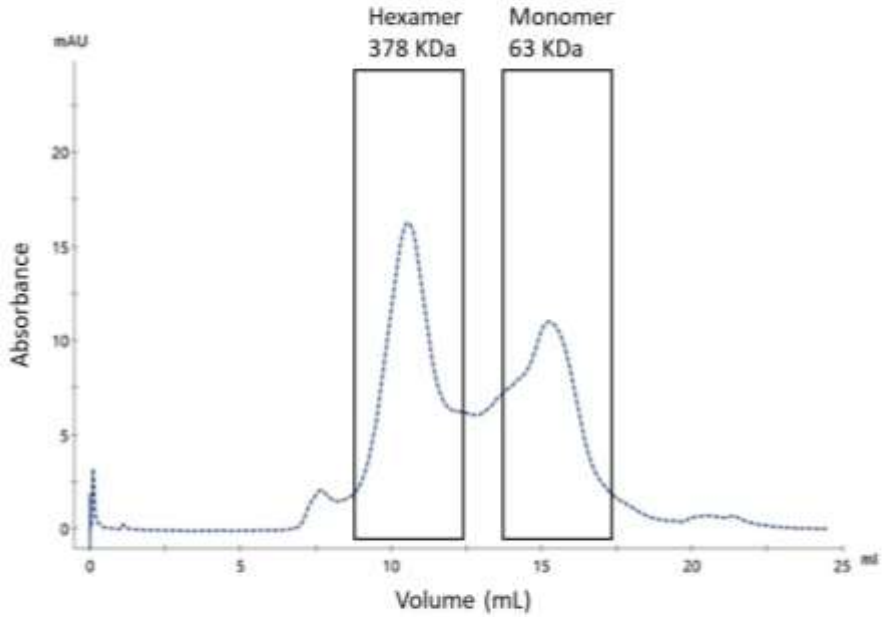

Figure S6: Size-exclusion chromatogram of the *Bs*GDH–*Tk*ADH fusion construct.
SEC analysis (A280) reveals multiple elution peaks corresponding to distinct oligomeric species, consistent with a heterogeneous population comprising monomeric hexameric assemblies.

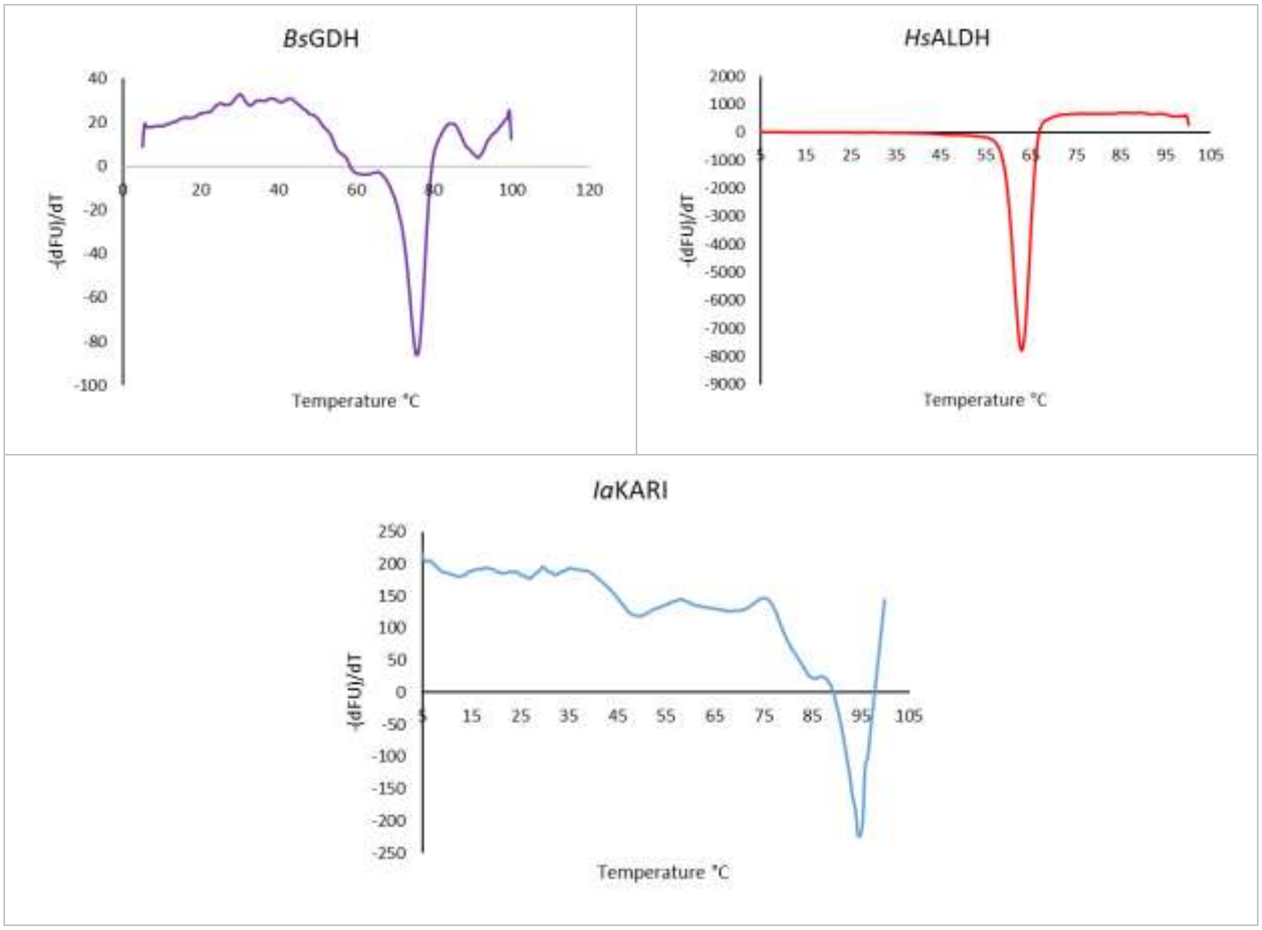

Figure S7: Thermofluor analysis of free enzymes. Thermal unfolding profiles of the individual oxidoreductases show high intrinsic thermostability (*Bs*GDH: 77.5 °C; *Hs*ALDH: 63 °C; *Ia*KARI: 94 °C). *Tk*ADH did not exhibit a detectable melting transition within the measured temperature range (<100 °C)

(i)

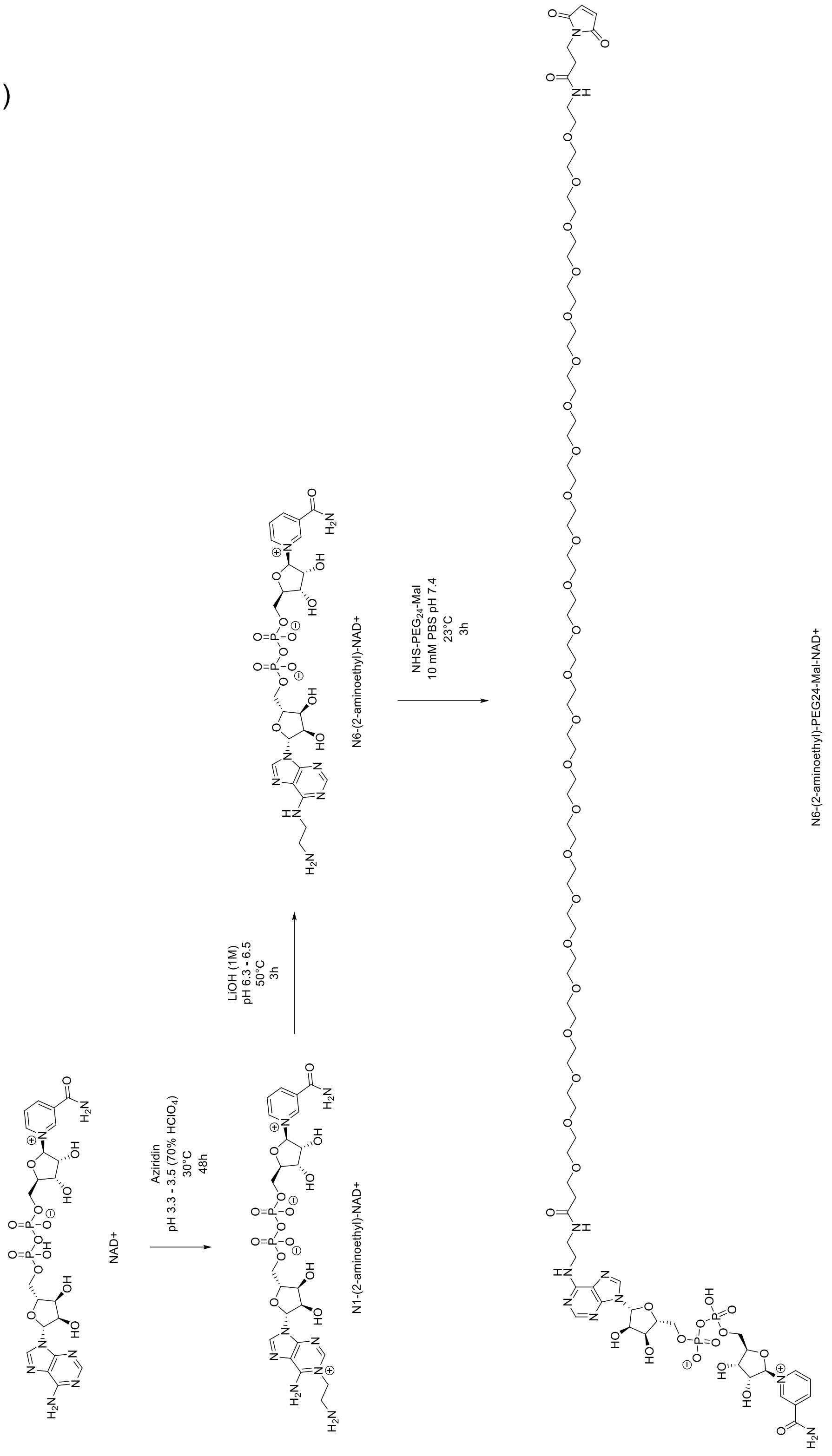
NAD+
Aziridin
pH 3.3 - 3.5 (70% HClO4)
30°C
48h
N1-(2-aminoethyl)-NAD+
LiOH (1M)
pH 6.3 - 6.5
50°C
3h
N6-(2-aminoethyl)-NAD+
NHS-PEG24-Mal
10 mM PBS pH 7.4
23°C
3h
N6-(2-aminoethyl)-PEG24-Mal-NAD+

(ii)

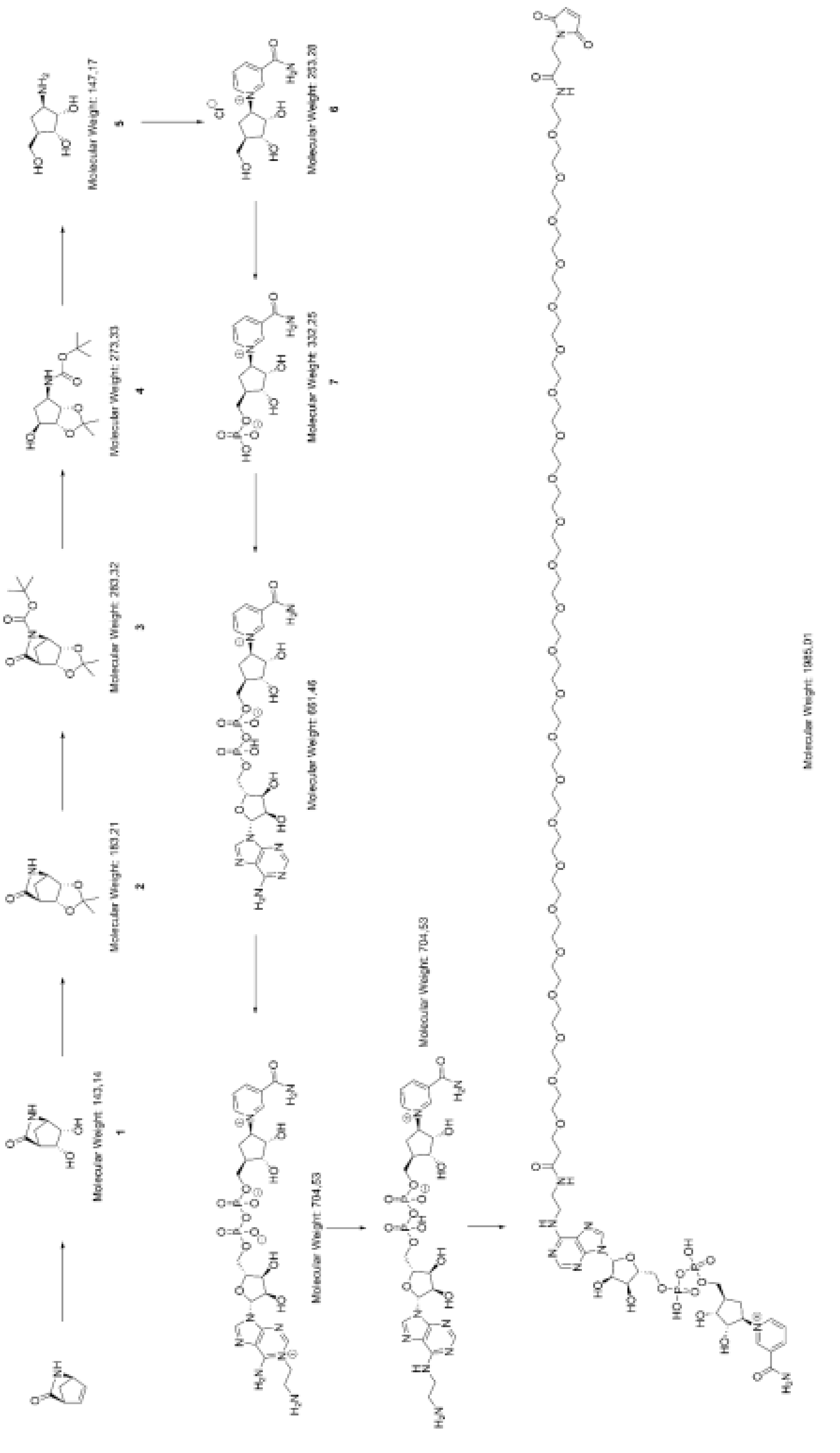

Figure S8: Synthesis scheme for the modified cofactor Mal-PEG$_{24}$-N6-(2-aminoethyl)-NAD$^+$.

Synthesis of ii) Mal-PEG$_{24}$-N6-(2-aminoethyl)-cNAD$^+$

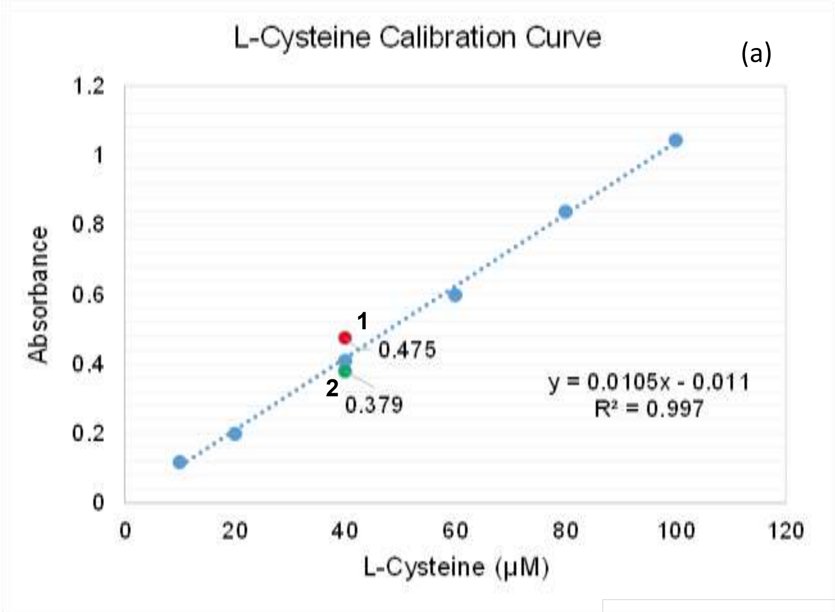

1. ***Tk*ADH-PEG-NAD$^+$-*Bs*GDH**

2. ***Ia*KARI-PEG-NAD$^+$-*Hs*ALDH**

(b)

Concentration (μM)	Absorbance	Comments
10	0.118	Standards (L-cysteine)
20	0.199	
40	0.409	
60	0.598	
80	0.838	
100	1.044	
40 [1] (before conjugation)	0.475	Fusion Enzymes
40 [2] (before conjugation)	0.379	
40 [1] (After Conjugation)	0.042	
40 [2] (After Conjugation)	0.049	

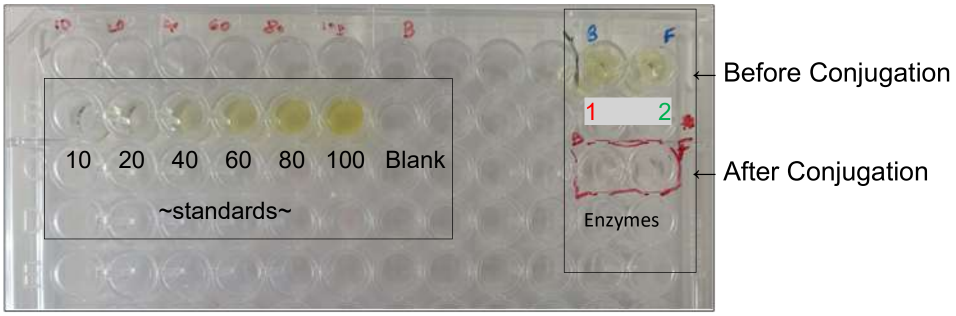

Figure S9: Validation of PEG–NAD$^+$ tethering by thiol reactivity (Ellman's) assay. (a) Calibration curve with L-cysteine standards (absorbance at 412 nm), with the free enzyme prior to conjugation corresponding to ~40 μM accessible thiols. (b) Quantification of free cysteine concentration before and after conjugation shows complete loss of reactivity toward DTNB,

consistent with full modification of the linker cysteine. (c) Microtiter plate assay illustrating the disappearance of the yellow DTNB color after conjugation, confirming covalent tethering of PEG24–NAD^+.

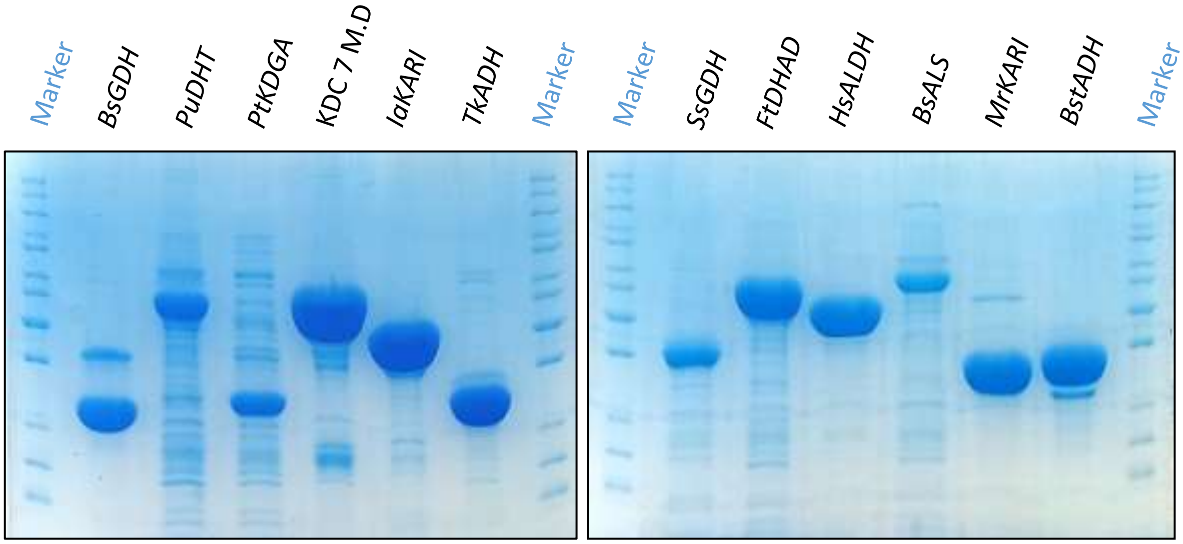

Figure S10 : SDS-PAGE analysis of purified enzymes used in this study. Each lane corresponds to a single enzyme preparation as indicated above the gel.

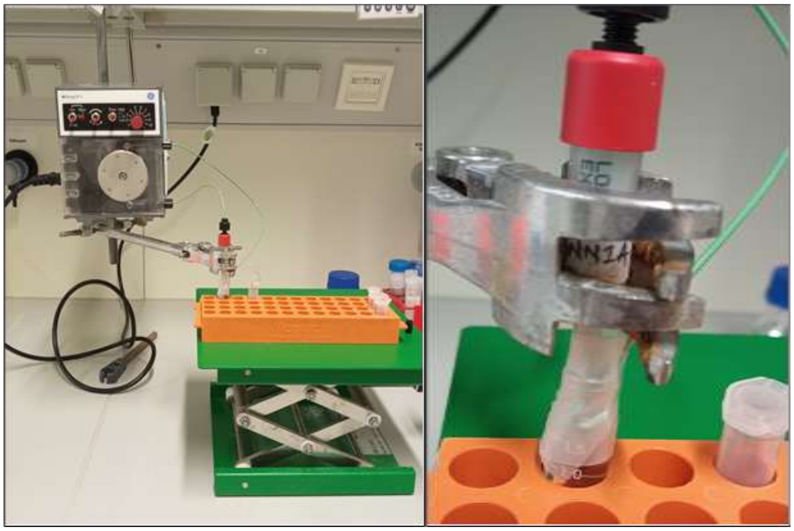

Figure S11: Continuous-flow setup for the glucose-to-isobutanol cascade. Photographs showing the experimental arrangement used for flow operation. Enzymes were immobilized on a 1 mL HisTrap column via N-terminal His-tags. The reaction mixture containing the substrate was circulated through the column using a peristaltic pump. Reservoir, pump, and column are connected in a closed loop as depicted.

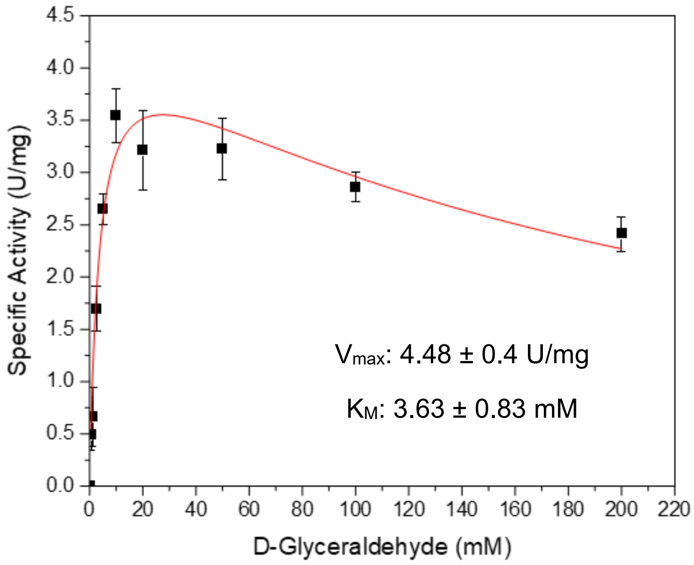

Figure S12: Kinetics of *Tk*ADH with D-Glyceraldehyde. Initial reaction rates (U/mg) were measured at varying glyceraldehyde concentrations (0-200 mM) in 50 mM HEPES, pH 7.5, RT) with saturating NAD^+. Data points represent means ± SD of triplicates. Rates were fitted to the Michaelis–Menten model incorporating substrate inhibition, $y = V_{max}. x / (K_m + x(1+x/K_i))$.

A) Standard 1

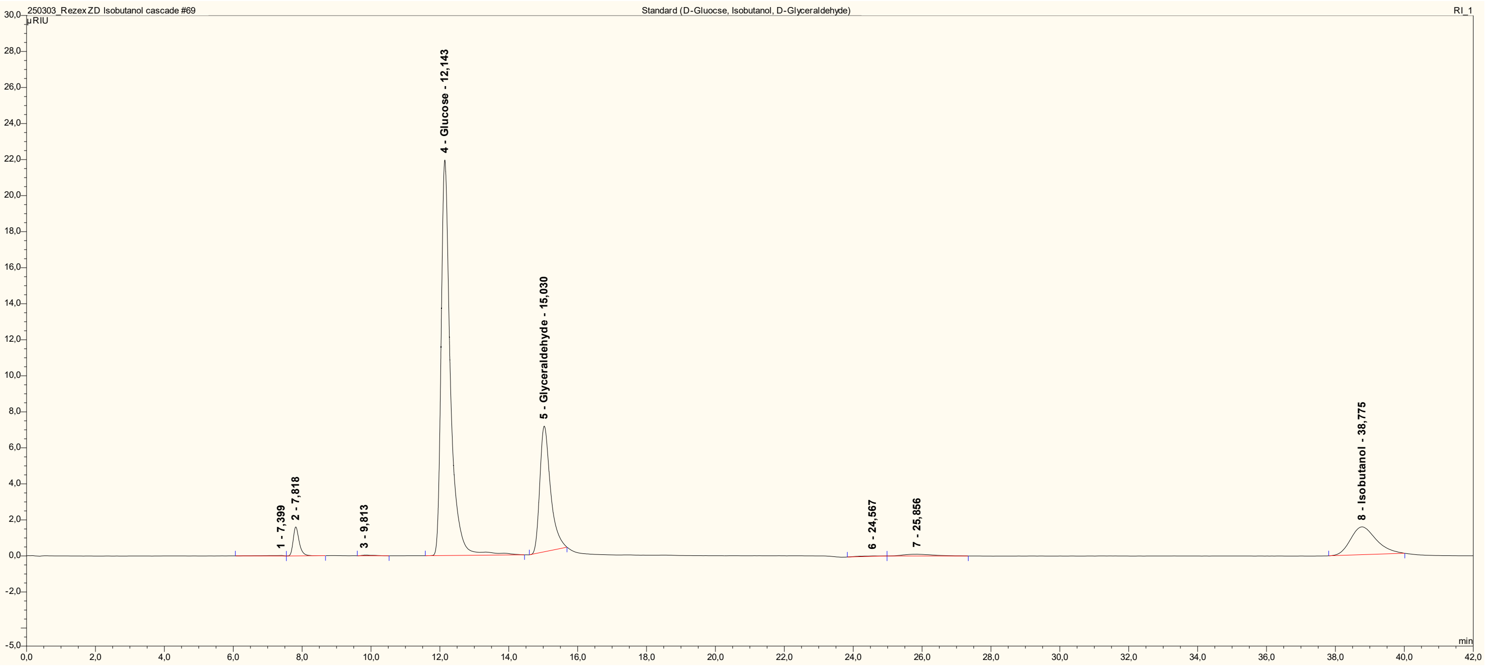

B) Standard 2:

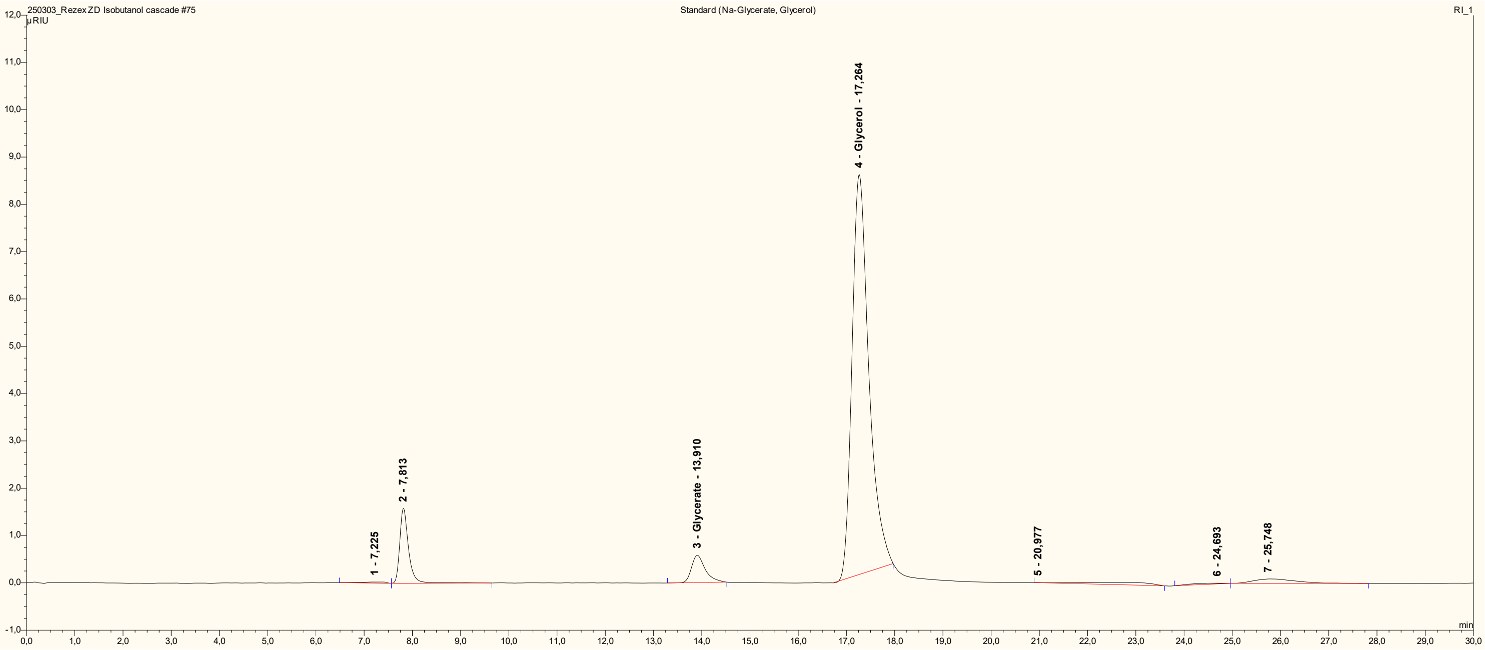

Figure S13: HPLC chromatograms of authentic standards used for compound identification and quantification. (A) Standard 1 mixture containing D-glucose (Retention time, tR = 12.14 min), D-glyceraldehyde (tR = 15.00 min), and isobutanol (tR = 38.77 min). (B) Standard 2 mixture containing sodium glycerate (tR = 13.91 min) and glycerol (tR = 17.26 min). Compounds were detected and quantified using a refractive index (RI) detector.

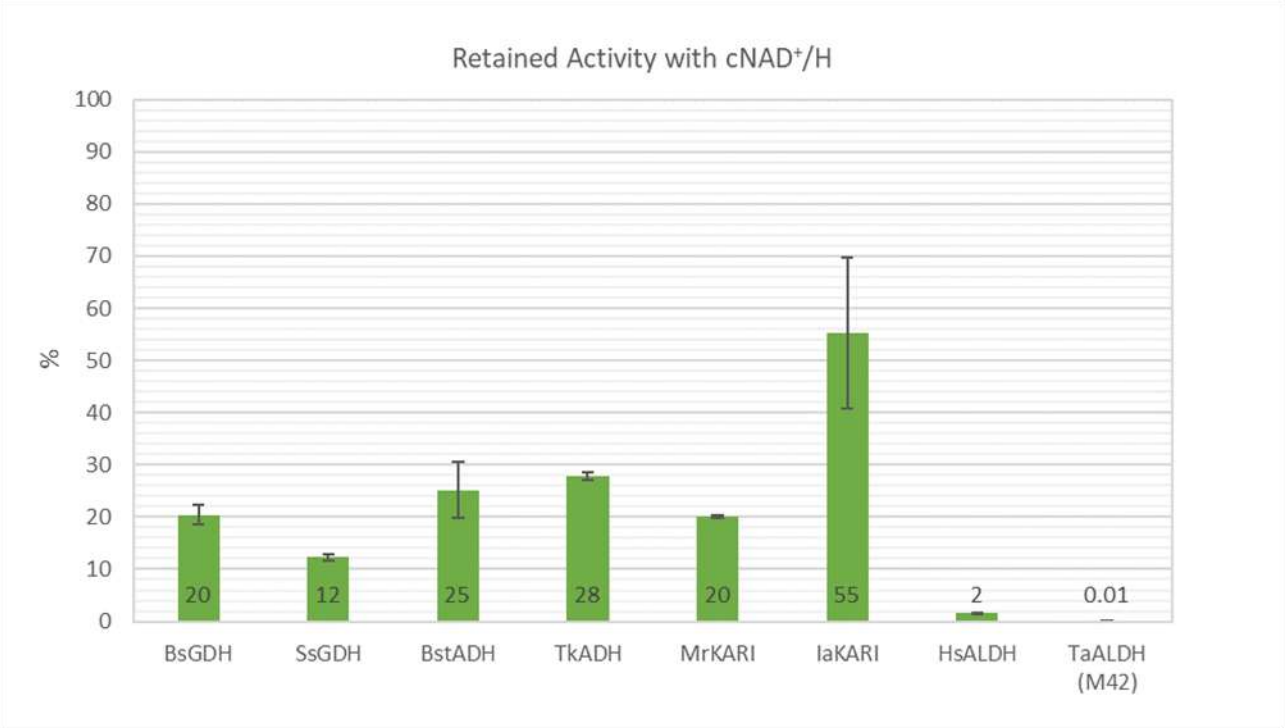

Figure S14: Retained activities of oxidoreductases with cNAD(H) relative to NAD(H). Initial-rate activities were measured under identical assay conditions using either NAD(H) or cNAD(H). Activities with NAD(H) were set to 100%, and activities with cNAD(H) are reported as retained activity.

Supplementary References

1. Melse, O. et al. Structure-Guided Modulation of the Catalytic Properties of [2Fe−2S]-Dependent Dehydratases. *ChemBioChem* **23**, e202200088 (2022).
2. Andreas F. Bückmann, V.W., Henk C. van der Plas Simultaneous Conversion of N(1)-(2-Aminoethyl)adenosine to N6-(2-Aminoethyl)adenosine and Tricyclic 1. N6-Ethanoadenosine under Mild Aqueous Conditions. *HETEROCYCLES* **41** (1995).
3. Bückmann, A.F. A New Synthesis Of Coenzymically Active Water-Soluble Macromolecular Nad And Nadp Derivatives. *Biocatalysis* **1**, 173-186 (1987).
4. Hartley, C.J. et al. Engineered enzymes that retain and regenerate their cofactors enable continuous-flow biocatalysis. *Nature Catalysis* **2**, 1006-1015 (2019).
5. Dsouza, Z. et al. Synthesis of thermostable artificial nicotinamide cofactors: carba-NAD+ and carba-NADP+. *RSC Chemical Biology* (2026).
